# BET protein inhibition sensitizes glioblastoma cells to temozolomide treatment by attenuating *MGMT* expression

**DOI:** 10.1038/s41419-022-05497-y

**Published:** 2022-12-13

**Authors:** Alessandro Tancredi, Olga Gusyatiner, Pierre Bady, Michelle C. Buri, Rémy Lomazzi, Davide Chiesi, Mahmoud Messerer, Monika E. Hegi

**Affiliations:** 1 Neuroscience Research Center, Lausanne University Hospital and University of Lausanne, Lausanne, Switzerland; 2 Service of Neurosurgery, Lausanne University Hospital and University of Lausanne, Lausanne, Switzerland; 3 Translational Data Science & Biomedical Data Science Center, Lausanne University Hospital and University of Lausanne, Lausanne, Switzerland; 4 SIB Swiss Institute of Bioinformatics, Lausanne, Switzerland

**Keywords:** CNS cancer, Mechanisms of disease

## Abstract

Bromodomain and extra-terminal tail (BET) proteins have been identified as potential epigenetic targets in cancer, including glioblastoma. These epigenetic modifiers link the histone code to gene transcription that can be disrupted with small molecule BET inhibitors (BETi). With the aim of developing rational combination treatments for glioblastoma, we analyzed BETi-induced differential gene expression in glioblastoma derived-spheres, and identified 6 distinct response patterns. To uncover emerging actionable vulnerabilities that can be targeted with a second drug, we extracted the 169 significantly disturbed DNA Damage Response genes and inspected their response pattern. The most prominent candidate with consistent downregulation, was the O-6-methylguanine-DNA methyltransferase (*MGMT*) gene, a known resistance factor for alkylating agent therapy in glioblastoma. BETi not only reduced *MGMT* expression in GBM cells, but also inhibited its induction, typically observed upon temozolomide treatment. To determine the potential clinical relevance, we evaluated the specificity of the effect on *MGMT* expression and MGMT mediated treatment resistance to temozolomide. BETi-mediated attenuation of *MGMT* expression was associated with reduction of BRD4- and Pol II-binding at the *MGMT* promoter. On the functional level, we demonstrated that ectopic expression of *MGMT* under an unrelated promoter was not affected by BETi, while under the same conditions, pharmacologic inhibition of MGMT restored the sensitivity to temozolomide, reflected in an increased level of γ-H2AX, a proxy for DNA double-strand breaks. Importantly, expression of MSH6 and MSH2, which are required for sensitivity to unrepaired O6-methylguanine-lesions, was only briefly affected by BETi. Taken together, the addition of BET-inhibitors to the current standard of care, comprising temozolomide treatment, may sensitize the 50% of patients whose glioblastoma exert an unmethylated *MGMT* promoter.

## Introduction

New avenues have to be taken to improve the outcome of patients with glioblastoma (GBM) who have a median survival of <2 years. No major improvements have been made since 2005, when TMZ was introduced [1], despite numerous efforts with targeted agents or immunotherapies that have shown some efficacy in other solid tumors. The striking failures of these single agent therapies [2] have incited the exploration of rational combination therapies that synergistically induce tumor vulnerabilities, sensitizing the cells to treatment. The development of drugs targeting epigenetic modifiers, such as Bromodomain and extra-terminal tail (BET) proteins, holds new opportunities [3, 4]. Overexpression of proto-oncogenes in cancer has been associated with increased binding of BET proteins to their promoter region and respective active enhancer elements [5]. BET proteins are epigenetic readers that recognize acetylated lysines on histone tails and recruit proteins to the transcriptional complex, thereby connecting the histone code to gene transcription. This interaction can be targeted by small-molecule BET inhibitors (BETi) that specifically bind to the tandem domains of BET proteins and strip BET proteins from the chromatin, thereby inhibiting gene expression [5]. Treatment of cancer cells with BETi, such as the tool drug JQ1, disturb cancer relevant pathways that may uncover vulnerabilities targetable with a second drug as we and others have reported previously [6, 7].

Based on the fact that genotoxic treatments show some efficacy in GBM, such as combined chemo-radiotherapy with the alkylating agent temozolomide (TMZ), the current standard of care [8], we set out to uncover potential BETi-induced vulnerabilities in the DNA damage response (DDR). Previous work, reporting on opportunities to target DDR in cancer, provides a valuable resource to identify potentially synergistic drugs [9].

Here, we report on the potential of BETi to modulate the DDR in GBM cells, including the gene that encodes the O6-methylguanine-DNA methyltransferase (*MGMT)*. MGMT expression is a known resistance factor to TMZ treatment [10] as it repairs the most toxic lesion, O6-methylguanine, thereby blunting the treatment effect [11]. We demonstrate that BETi specifically down regulates endogenous *MGMT* expression in GBM cells, sensitizing them to TMZ therapy, without compromising the mismatch repair (MMR) system that is essential for sensitivity to alkylating agent therapy [12, 13]. These findings provide evidence that the addition of BETi in combination with TMZ may overcome treatment resistance in patients, whose GBM harbor an unmethylated *MGMT* promoter by directly inhibiting *MGMT* expression.

## Material and methods

### Clustering procedure for trajectory analysis of RNA-seq data and time

The RNA-seq data reported in Gusyatiner et al. [6] served as input and was obtained in the GBM derived sphere line LN-2683GS upon treatment with 1 μM JQ1 or DMSO over a time course of 48 h with three biological replicates. Differential gene expression analysis used a model with full interaction between treatment and time (edgeR package). Genes significantly associated with JQ1-treatment were identified, based on Bonferroni corrected *p*-values (FWER), derived from log-likelihood ratio test for generalized linear models (GLM) and averaged log2-counts per millions (CPM) by gene as measure for expression level. This selection yielded 4712 genes (FWER < 0.1 and log_2_(CPM + 1) > 1) [6]. Afterwards, their temporal trajectories were classified in function of their response pattern to the treatment by a two-step procedure as illustrated in a flow-chart in Supplemental Fig. S1. The first step consisted of randomly selecting 500 genes to establish the optimal number of temporal patterns. The Fréchet distance [14, 15] was used to compare the trajectories of the genes. To reduce the data noise Principal coordinate analysis was performed (PCO [16]) on a pairwise distance matrix. The three first components of the PCO were then used to partition the 500 selected genes by K-means clustering. Several partitions forming a cascade from a small (*k* = 2) to a large number (*k* = 10) of groups were created. The optimal number of clusters was defined using the Calinski-Harabasz (*Calinski’s*) criterion [17]. Averaged profiles (reference profiles) were computed for each cluster. In the second step, the *re-clustering* of all genes was provided by the computation of the Fréchet distance between each averaged profile and each gene. The corresponding cluster was attributed according to the minimal distance to the reference/averaged profiles. Gene set enrichment analyses (GSEA) were based on hypergeometric tests, associated with Bonferroni correction of the *p*-value for multiple testing. GSEA were performed by cluster for DDR pathways defined by Pearl et al. [9]. All analyses (e.g. “cascade” K-means and Fréchet distance), differential expression analysis, enrichment analysis and graphical representation related to longitudinal clustering were performed in R (URL http://www.R-project.org) [18] and the R packages vegan, longitudinalData, edgeR, clusterProfiler and ade4.

### Cell culture

Patient-derived GBM sphere (GS) lines LN-2683GS and LN-4372GS, and the adherent cell lines LN-18, LN-229, LN-340, and LN-382 were established and molecularly characterized in our laboratory [19–21] according to institutional directives, approved by the Ethics Committee of the Canton de Vaud (CER-VD, protocol F25/99). T98G was obtained from ATCC and the GBM Glioma Stem Cell (GSC) line MDA-GSC-23 (GSC23, RRID:CVCL_DR59), with and without a luciferase-construct, was obtained under a Material Transfer Agreement from the University of Texas MD Anderson Cancer Center (Houston,TX). Methylome data (EPIC BeadChip) for MDA-GSC-23 are available at the Gene Expression Omnibus database (GEO, http://www.ncbi.nlm.nih.gov/geo/) under the accession number GSE217515. The *MGMT* promoter methylation status is summarized in Supplemental Table S1A. All lines were regularly tested mycoplasma-free (MycoAlert Kit Lonza, Cat. LT07-418), and were authenticated in 2022 by STR fingerprinting at the Forensic Genetics Unit of the University Center of Legal Medicine, Lausanne and Geneva [22]. The STR profile for the recent glioma sphere line LN-4372GS is reported in Supplemental Table S1B. Adherent cell lines were grown in Dulbecco’s modified Eagle medium (DMEM, Glutamax Gibco™, Life Technologies, Cat. 61965-026) with 5% Fetal Bovine Serum (Hyclone). Glioma sphere (GS) / glioma stem cell (GSC) lines were maintained under neural stem cell culture conditions in DMEM/F12 (Life Technologies, Cat. 31331-028) containing B27 supplement and growth factors, as previously described [23]. The transduced cell lines were continuously maintained under respective selection. BET inhibitors were dissolved in DMSO at 10 [mM] and added to the cells at the concentrations indicated (JQ1, APExBIO, No. A1910; I-BET, ODM-207, Orion pharmaceuticals).

### Molecular cloning

LN-229MGMT^ind^_C12 was derived from LN-229 upon transduction with a Tet-ON inducible system for *MGMT* as previously described [24], and maintained under Blasticidin (Thermofisher, R21001) selection at 10 [µg/ml]. *MGMT* was induced with doxycycline (Dox, Sigma Aldrich, D9891-1G) at 100 [ng/ml].

### Production and delivery of lentiviral particles

For the production of LN-340shMSH6#1^ind^_C8 and LN-340shMSH6#2^ind^, and the respective LN-340shCTRL^ind^, we obtained TRIPZ Dox-inducible Lentiviral shRNA targeting h*MSH6* as E. coli glycerol stock cultures (Horizon Discovery Ltd. Clone Id: V2THS_258239 & Clone Id: V2THS_82749), and the non-targeting TRIPZ shRNA designed with minimal homology to known mammalian genes (Horizon Discovery Ltd. Catalog ID:RHS4743), respectively. The replication-incompetent lentiviral particles were produced according to the manufacturer’s protocol (Dharmacon™ Trans-Lentiviral packaging kits, Cat. TLP5912). After an incubation of 16 h the transfection mix was removed and 14 ml DMEM 5% FBS were added. After 48 h, the virus containing supernatant was harvested, passed through a 0.22 µM filter and complemented with 10 [µg/ml] Polybrene (Sigma-Aldrich, TR-1003-G), and added to the target cells for 24 h. The medium was replaced with fresh DMEM 5% FBS, and after 24 h incubation cells were subjected to selection by adding 5 [µg/ml] Puromycin (Catalog Number P8833, Sigma-Aldrich). Expression of the shRNAs were induced by treatment with Dox at 500 [ng/ml].

### RNA extraction and qRT-PCR

Total RNA isolation and qRT-PCR were performed as described previously [23] using primers compiled in Supplemental Table S2. The expression levels were normalized to *GAPDH*.

### Protein extraction and western blot

Cells were collected by centrifugation for GS-lines and by scraping for adherent cells. Westerns were done as described [23] and probed with respective antibodies: anti-α-Tubulin (Sigma, T5168, 1:10,000), anti- β-Actin (Bioconcept, 8H10D10. 1:10,000), anti-MGMT (R&D systems, AF3794-SP, 1:4000), anti-MSH6 (Cell Signaling, #5424 S, 1:4000), anti-MSH2 (Cell Signaling, #2017S, 1:4000). Membranes were washed 5 min x3 in TBS-T at RT, followed by incubation at RT for 1 h with the following secondary antibodies (according to primary antibody specifics): anti-rabbit (Promega, W4011, 1:5000), anti-mouse (Thermofisher, 31430, 1:5000), anti-goat (Thermofisher, 31402, 1:5000).

### Chromatin immunoprecipitation (ChIP)-qPCR

ChIP-qPCR was largely performed following the iDeal ChIP-seq kit for Transcription Factors (Diagenode, cat. C01010170). Briefly, proteins from 20 M T98G cells were cross-linked to DNA in a 15 cm petri dish by adding fresh Paraformaldehyde (PFA) (Lucerna, cat. 15714) to a final concentration of 1% for 15 min at RT. Fixation was quenched with Glycine for 5 min at RT. Fixed cells were then washed with cold PBS, and nuclei were extracted via cell membrane lysis. Using 1.5 ml Bioruptor^®^ Microtubes with caps (Diagenode, cat. C30010016), chromatin was sonicated at a density of 1.5 M cells/ 100 µl complete Shearing buffer iS1b with Bioruptor Pico (Diagenode, Serial Number P-181503) for 12 cycles (30 s “ON”, 30 s “OFF”) in order to obtain fragments between 100 bp and 600 bps. The chromatin was briefly centrifuged for 15 s, and subsequently, the supernatant was centrifuged for 10 min at 4 °C at 16,000 g. An aliquot of 50 µl of the supernatant was kept for shearing assessment, and the sample was stored at -80 °C for subsequent immunoprecipitation. The chromatin was decross-linked with 1 µl of proteinase K 20 [mg/ml] (Life Technologies, cat. AM2546) overnight at 65 °C. DNA was extracted by adding one volume of phenol/chloroform/isoamyl alcohol (25/24/1) to the sample and mixed vigorously for 30 s. Samples were centrifuged at RT for 5 min at 16,000 g. The aqueous phase was carefully removed and transferred to a new tube. 2.5 volumes of ice-cold 100% ethanol, 0.5 volumes of sodium acetate and 1 µl of GlycoBlue™ Coprecipitant (Invitrogen, cat. AM9515) were added to the samples and incubated at -80 °C for 2 h. Samples were centrifuged for 30 min at 4 °C at 16,000 g. The supernatant was carefully removed and the DNA pellet was washed with 300 µl 70% ethanol. Samples were centrifuged for 10 min at 4 °C at 16,000 g. The supernatant was removed, and the DNA pellet was air-dried. The pellet was dissolved in 30 µl TE elution buffer and DNA was quantified with the Qubit™ dsDNA HS Assay Kit (Thermofisher, cat. Q32851). 300–600 ng of DNA were analyzed on a 1.5% agarose gel to determine fragment sizes. Samples with fragments between 100 bp and 600 bps were used for subsequent magnetic immunoprecipitation. Frozen sheared chromatin pellets were incubated overnight at 4 °C under constant rotation with the corresponding ChIP reaction mix. Each ChIP reaction mix corresponds to 1 immunoprecipitation of interest: anti-BRD4 (Bethyl Laboratories Inc., A301-985A50, 2 µg/IP), anti-Pol II (Cell Signaling Technology, CST14958, 1 µg/IP), anti-CTCF (Diagenode Kit, 2 µg/IP), anti-IgG (Diagenode Kit, 1 µg/IP). Subsequently, immunoprecipitated DNA was eluted, decross-linked, and purified according to protocol. DNA was quantified with Qubit™ dsDNA HS Assay Kit for quality control purposes only. Shearing assessment was performed by adding 50 µl TE elution and RNAse A (Thermo Fisher Scientific, cat. EN0531) for 1 h at 55 °C. Immuno-precipitated DNA and corresponding INPUT were analyzed by qPCR analysis with primers of interest [19, 25] (Supplemental Table S2). Finally, the relative amount of immune-precipitated DNA compared to INPUT DNA (% of recovery) was calculated.

### Immunofluorescence analysis for γ-H2AX and data processing

Target cells were seeded on open µ-Slides (chambered coverslip) with 8 wells (Vitaris, 80826) (between 2500–3000 cells/well). Cells were treated with JQ1 and Dox for 5 days prior TMZ (Sigma-Aldrich, T2577) and O6-Benzylguanine (O6BG; Sigma-Aldrich, 19916-73-5) treatments. Subsequently, cells were incubated for 48 h. Cells were fixed with 4% PFA (Lifetechnologies, cat. 28908) for 15 min at RT, followed by permeabilization with 0.3% Triton-X for 15 min at RT. Cells were blocked at RT for 1 h in blocking buffer (5% Donkey Serum, 0.5% BSA, 0.3% Triton-x-100). Cells were incubated overnight at 4 °C with γ-H2AX AB (Cell signaling, 2577, 1:800 in blocking buffer). Secondary antibody Alex Fluor 647 (Thermofisher, A31573, 1:300 in blocking buffer) was added to cells for 1 h at room temperature. Before microscopy, DAPI was added and incubated for 15 min at RT. Image acquisition was performed with Zeiss LSM 880 Airyscan at 40x magnification with oil. Settings included 2 color channels/excitations; DAPI (408 nm, Blue) and P-H2AX/Alexa Fluor 647 (633 nm, Far red). Fifteen images per condition were acquired and further analyzed with the Cell Profiler software Version 3.1.9 (https://github.com/CellProfiler/CellProfiler/releases?page=4). γ-H2AX was quantified as integrated intensity using an optimized image acquisition software pipeline. In brief, the images acquired with confocal microscopy were exported as TIF files for Cell Profiler. A pipeline including metadata identity, object recognition, and calculation steps were optimized. Object recognition, nuclei, were identified and parameters optimized (nuclei size, 50–165 pixel; threshold, Global; threshold method, Otsu: two classes; threshold smoothing scale, 1.34; correction 1; bounds of threshold, 0.01 to 1.0; clumps object identity, shape). P-H2Ax served as input image, operation was set at enhanced, and the feature as speckles, feature size 10, and speed low. Object relation between nuclei (parent) and P-H2Ax (child), were obtained by per parent means for all child measurements (children per parent), and saved as RelateObjects (integrated intensity of P-H2Ax). After computation, the parameters of interest were selected and exported into excel format. From the excel file, the number of cells, and integrated intensity of P-H2Ax were used for analyses.

### Cell viability analysis

Cells were seeded into 48-well plates and treated for 5 days with JQ1, followed by three shots of TMZ at an interval of 6 h, using a clinically relevant dose of 100 μM, according to a previously reported schedule [26], and 1 shot of 10 μM O6BG. After 96 h, cells were stained with the CyQUANT Direct Cell Proliferation Assay Kit (Thermoscientific, cat. C35011). Following 1 h incubation, cells were scanned, and fluorescence was measured with the SpectraMax® M Series Multi-Mode Microplate Reader. For the experiments depleting MSH6 with the inducible sh-M*SH6*-constructs, cells were pretreated for 5 days with Dox at 500 [ng/ml], followed by the same treatment scheme as described above.

### Live-cell imaging of cell growth and cell death

LN-340 cells were seeded into a 96-well plate (3596, Corning) at a density of 730 cells/well Cells were pretreated with JQ1 or DMSO for 5 days, followed by the treatment schedule described under “Cell Viability” (DMSO, TMZ, O6BG, and combination). To monitor cell death, IncuCyte™ Cytotox Red Reagent (Essen BioScience, 4632) was added to the plate to a final concentration of 250 nM at the last treatment. The cells were then transferred into the Incucyte Zoom incubator and monitored taking images at a 10x magnification every 2 h for 70 h in phase contrast and Red channels. Phase contrast was used to determine cell proliferation (% of confluence), Cell death was monitored in the Red channel (Cytotox Red, total red object area Each value is the mean of three technical replicates). The experiments were repeated four times. Treatment with 20%DMSO or 5 μM Actinomycin D served as positive controls.

### Cell cycle analysis

LN-340 cells were seeded (0.25 M/10 cm petri dish per condition). Following 5 days pretreatment with JQ1 or DMSO, cells were treated with TMZ and O6BG. After 48 h cells were washed with PBS and fixed with ice-cold 70% ethanol overnight at 4 °C. Subsequently, cell pellets were washed with ice-cold PBS and treated with 1 ml Propidium Iodide solution (20 µg/ml final concentration) (Sigma-Aldrich, P4864-10ml) and RNAse A. Following at least 4 h incubation at 4 °C (protected from light), cells were filtered through 5 ml Round Bottom Polystyrene Test Tubes, with Cell Strainer Snap Cap (Falcon®). Stained and filtered cells were immediately processed with the Gallios II Beckman Coulter (Flow Cytometry Facility—University of Lausanne). Cell cycle distribution was analyzed with the FlowJo software.

### Statistical analysis

Statistical analysis of the experiments was executed using GraphPad Prism 9 Software. The responses to the treatment over the time course (48 h) were tested by two-way ANOVA, including the interaction term between time and treatment and using the Geisser-Greenhouse correction for variance heterogeneity. The analyses were completed by Dunnett’s multiple comparisons test as post hoc tests. The differences of means among the treatments, JQ1, TMZ and O6BG were tested by three-way ANOVA including the interactions between the treatments (first and second order interaction effects). Additive mixed model with interactions between treatments was used when the groups were unbalanced. The Tukey’s multiple comparisons tests were used as post hoc tests. Two-group comparison tests were performed by two-tailed ratio paired *t*-test including correction for variance heterogeneity. The comparison of several groups was provided by one-way ANOVA completed by Dunnett T3 multiple comparisons tests. Statistical significance was defined according to *p*-values, indicated by the asterisk symbol (*) in the Figures: (*) *p* < 0.05, (**) *p* < 0.01, (***) means *p* < 0.001. (****) means *p* < 0.0001. Data are shown as mean values. Error bars represent Standard Deviation (SD), unless indicated otherwise.

## Results

### BET protein inhibition disturbs DNA damage response signaling pathways in glioblastoma

**Fig. 1:**
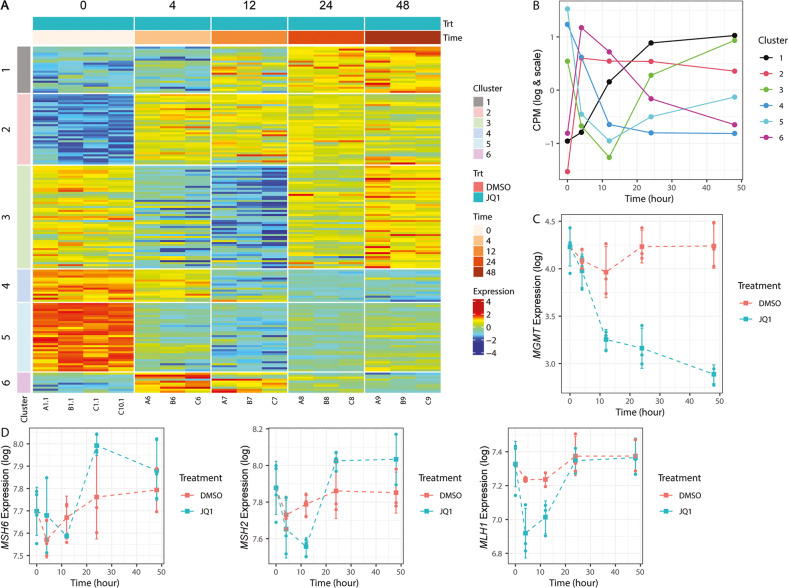
Response patterns of DDR genes in LN-2683GS cells treated with 1 µM (+)-JQ1 or DMSO for 4, 12, 24, and 48 h. The heatmap (**A**) illustrates the normalized expression of the DDR genes upon JQ1 treatment over a time course of 48 h. The gene trajectories were classified into the six clusters of response to the treatment defined by the two-step procedure based on Fréchet distances. **B** The six averaged expression patterns of DDR genes are displayed upon clustering over the time course of treatment. **C** The expression profile of the *MGMT* gene, classified into cluster 4, is represented in function of the time and stratified by tretament (JQ1 in blue and DMSO in red). **D** Similarly, the expression profiles of MMR genes *MSH6*, *MSH2* and *MLH1* upon JQ1 (red) or DMSO (blue) treatment are shown over the time course of 48 h, classified into cluster 3. The circles and squares correspond to the biological replicates and the means of the three independent biological replicates for each time point, respectively. The standard deviations (SD) are represented by the vertical lines.

In an effort to leverage clinically relevant pathways disturbed by BETi for druggable targets, we analyzed differential gene expression data obtained in a GBM-derived sphere line, LN-2683GS, treated with the tool drug JQ1. The cells treated with 1 μM JQ1 over a time course of 48 h underwent extensive transcriptome changes as we previously reported [6]. Significant association with JQ1-treatment was observed for 4712 genes (adjusted *p*-value by Bonferroni correction, <0.1 and log_2_(CPM + 1) > 1), whereof 169 were annotated as DDR genes as defined by Pearl et al. [9]. To identify JQ1-response patterns we determined the optimal number of gene clusters using K-means and obtained 6 clusters, as visualized in Supplemental Fig. S1 (*Calinski’s criterion* graphic and a corresponding heatmap). The comparison of the original K-means clustering and the re-clustering method exhibited a similar classification for the 500 training datasets. The percentage of good classification was equal to 95% with a kappa value of 0.94. Two clusters showed JQ1-induced gene expression over time (clusters 1 and 2), and two clusters displayed consistent downregulation (clusters 4 and 5), while two clusters displayed transient down- (cluster 3) or upregulation of expression (cluster 6), respectively. The 169 DDR genes, which are the focus of this study (Supplementary Table S3), were distributed among all six JQ1-response patterns (cluster 1, 23 genes; cluster 2, 35 genes; cluster 3, 51 genes; cluster 4, 16 genes; cluster 5, 34 genes; cluster 6 and 10 genes) as visualized in a heatmap (Fig. 1). Enrichment analyses of DDR pathways by cluster, showed only one significant association between cluster 3 and MMR genes (adjusted *p*-value by Bonferroni correction < 0.001). The annotated list of all 169 retained DDR genes, their expression levels by treatment and time point, cluster affiliation, Fréchet distance, and pathway information, based on Pearl et al. [9], is available in Supplementary Table S3.

A direct effect of BETi may be expected among rapidly downregulated genes by stripping BET proteins from their binding sites. Inspection of the clusters 4 and 5 revealed *MGMT* among the consistently downregulated genes, following expression pattern 4, as visualized in Fig. 1. The consistent downregulation by JQ1, identified *MGMT* as a prime target which opens the opportunity to sensitize GBM with an unmethylated *MGMT* promoter to TMZ. Patients with *MGMT* unmethylated GBM basically show no benefit from TMZ therapy [10]. To exclude a JQ1-specific effect, we confirmed inhibition of endogenous *MGMT* expression using two other BET inhibitors, I-BET and ODM-207, in LN-2683GS and LN-18 (Supplemental Fig. S2).

Given the fundamental role of a fully functional MMR system for sensitivity of cells to O6-methylguanine lesions in MGMT-deficient cells, we paid attention to the modulation of key genes involved in the MMR system. As mentioned above, the MMR genes were significantly enriched in cluster 3, and comprised among others *MSH6*, *MSH2* and *MLH1* that have been associated with acquired treatment resistance to TMZ in recurrent gliomas when mutated or silenced otherwise [27]. Even though the expression of *MSH6*, *MSH2* and *MLH1* was transiently downregulated upon JQ1 treatment at early time points (6 h and 12 h), it was restored to baseline 24 h after initiation of JQ1-treatment, suggesting that BETi does not compromise MMR in this sphere line (Fig. 1, Supplementary Table S3).

### BET protein inhibition reduces *MGMT* expression and prohibits its induction upon temozolomide treatment

**Fig. 2:**
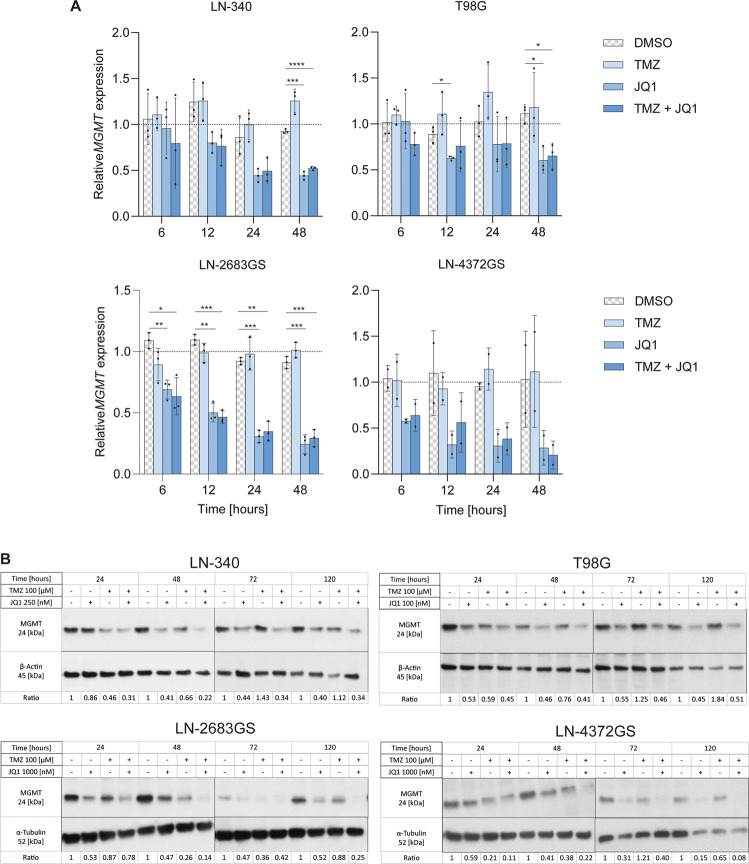
BET protein inhibition reduces MGMT expression in GBM. **A** qRT-PCR analysis of relative *MGMT* gene expression at 6, 12, 24 and 48 h after treatment with [100 μM] TMZ and JQ1 at the following concentrations: [250 nM] for LN-340, [100 nM] for T98G, and [1000 nM] for the sphere lines LN-2683GS and LN-4372GS. Each data point represents an independent experiment. Data were normalized to the respective DMSO treatment for each time point (baseline). Adjusted *p*-values (*p*) were determined by Dunnett’s multiple comparisons test following two-way ANOVA using Geisser-Greenhouse correction for variance heterogeneity and including interaction between time and treatment. Error bars are SD. *(*p* ≤ 0.05), **(*P* ≤ 0.01), ***(*P* ≤ 0.001). **B** Protein expression analysis of MGMT, β-Actin and α-Tubulin by western blot, 24, 48, 72 and 120 h after treatment. One of three representative biological replicates is shown. The quantification of three replicates is shown in Supplemental Fig. S2B. Full length western blots are available in Supplemental Material.

Next, we monitored downregulation of *MGMT* in several GBM sphere and cell lines with endogenous MGMT expression on the RNA and protein level in response to treatment with JQ1, TMZ, and their combination. The JQ1 concentrations were adapted to the sensitivity of the individual cell and sphere lines (0.1–1 μM), and TMZ was used at a clinically relevant dose of 100 μM [26, 28]. The *MGMT* expression was significantly affected by treatment over the time course of 48 h for the cell lines LN-340, T98G, and LN-2683GS, the same trend was observed for LN4372GS, but did not reach significance, as visualized in Fig. 2A (Supplemental Table S4).

The results confirmed rapid downregulation of *MGMT* expression upon JQ1 treatment as measured over a time course of 48 h (Fig. 2A). Moreover, *MGMT*-induction generally observed upon TMZ treatment alone, was prohibited by JQ1, and the expression levels were kept significantly below the baseline over the time course. This behavior also translated to the protein level, although with a delay (Fig. 2B, Supplemental Fig. S3). Substantial MGMT depletion with JQ1 treatment alone was observed after 72 h of treatment, with a more pronounced effect after 120 h. TMZ treatment alone also showed a decrease of MGMT protein after 24 h, compatible with the suicide reaction of MGMT after transfer of the methyl group that leads to ubiquitination and proteasome-mediated degradation, requiring *de novo* synthesis [29]. These results were consistent across all the different GBM cell lines and sphere lines tested (Fig. 2). In the following experiments testing TMZ-related effects, cells were pretreated for 120 h with JQ1 (or DMSO, control) to allow for JQ1-mediated depletion of MGMT. It is of note, JQ1 alone will induce some apoptosis (PARP cleavage) towards the end of this pretreatment period, at 72 h and 120 h as illustrated for LN-340 (Supplemental Fig. S4) and as we reported previously for GBM sphere lines [6].

### BET protein inhibition reduces BRD4 occupancy at the *MGMT* promoter region

**Fig. 3:**
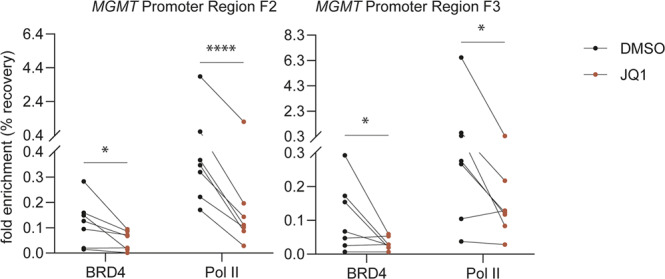
BRD4 occupancy at the *MGMT* promoter region is reduced upon JQ1 treatment. T98G cells were treated for 2 h with or without [1 µM] JQ1. ChIP-qPCR for BRD4 and Pol II occupancy in the promoter region of *MGMT* interrogated at both F2 and F3 regions are represented as enrichment (%input). The experiment includes seven independent experiments. *P*-values were determined by two-tailed ratio paired *t*-test including correction for variance heterogeneity. *(*p* < 0.05), ****(*p* < *P* ≤ 0.0001).

To investigate whether *MGMT* expression is directly regulated by BRD4, we performed chromatin immuno precipitation followed by quantitative PCR (ChIP-qPCR) analysis for BRD4 binding in the *MGMT* promoter region. T98G cells that exert relatively high levels of endogenous MGMT on the RNA and the protein level (Supplemental Fig. S5), were treated for 2 h with JQ1 [1 µM]. Cells were harvested and subjected to chromatin immunoprecipitation with anti-BRD4 antibodies. The relative BRD4 occupancy at the *MGMT* promoter was determined by ChIP-qPCR using previously described primer sets [19, 25]. The two regions interogated are located within the CpG island of the promoter and the first exon. The analysis demonstrated a significant difference in BRD4 occupancy at both *MGMT* promoter regions tested (both locations, F2, F3, *p* < 0.5, two-tailed ratio paired *t*-test, with correction for variance heterogeneity, Fig. 3). The JQ1-associated decrease in BRD4 binding was supportive of a direct regulatory effect on *MGMT* expression. Furthermore, we also evaluated associated changes of RNA polymerase II (Pol II) occupancy, a marker for active transcription. In line with the observed decrease in *MGMT* expression, we detected a significant difference in Pol II occupancy (F2, *p* < 0.0001; F3, *p* = 0.0377), with decreased binding at both *MGMT* promoter regions tested, suggesting attenuation of the *MGMT* transcription process.

Overall, our results support that *MGMT* transcription is attenuated upon JQ1 treatment due to BRD4 depletion at the promoter region.

### BET protein inhibition modulates TMZ-induced DNA damage in an MGMT-dependent manner

**Fig. 4:**
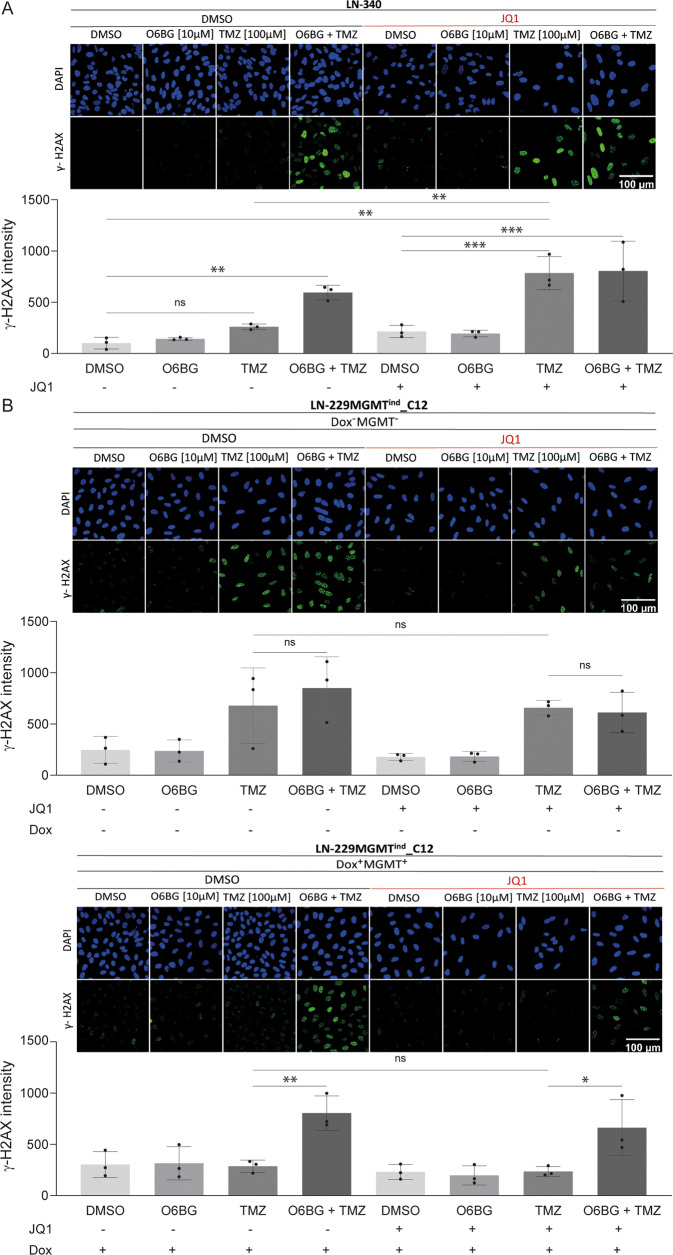
JQ1 modulates repair of TMZ-induced DNA damage. **A** Mean γ-H2AX integrated intensity analysis was performed on LN-340 cells using immunofluorescence (IF). Cells were pretreated with JQ1 [0.250 µM] for 5 days. On day 5, cells were treated with O6BG [10 µM] together with TMZ [100 µM]. Two additional TMZ treatments were given every 6 h, for a total of 3 TMZ treatments on day 5. End-point was set at 48 h after TMZ treatments. Data represent the mean from three independent biological experiments. The adjusted *p*-values were provided by Dunnett T3 multiple comparisons tests following one-way ANOVA. *(*p* ≤ 0.05), **(*p* ≤ 0.01), ***(*P* ≤ 0.001). Error bars are SD. **B** Cells of GBM line LN-229MGMT^ind^_C12 were treated according to the same schedule as in (**A**), but at a lower JQ1 concentration [100 nM]. The experiments were performed in absence and presence of doxycycline [100 ng/ml] (Dox), respectively. Dox treatment induces ectopic expression of *MGMT* under the control of the Tet-ON promoter. Scale bar 100 μm.

In light of the postulated direct downregulation of *MGMT* upon BETi in GBM lines, we investigated the role of BETi in modulating the TMZ-induced DNA damage response. We treated the MGMT expressing GBM cell line LN-340 with TMZ alone or in combination with JQ1 [0.25 μM], and monitored γ-H2AX levels, a marker for DNA double-strand breaks (DSBs) [30]. The formation of γ-H2AX foci is among the first steps that initiates the recruitment of DNA repair proteins. Cells were pretreated for 120 h with JQ1 or DMSO to allow for JQ1-mediated depletion of the MGMT protein, and γ-H2AX was measured 48 h after treatment with TMZ, quantifying immunofluorescence determined by confocal microscopy. In absence of JQ1, we observed that LN-340 showed no difference in γ-H2AX levels upon TMZ treatment alone, as compared to DMSO control, while a significant difference was observed upon treatment with the MGMT-specific pharmacologic inhibitor O^6^-benzylguanine (O6BG) [26, 31, 32] (adjusted *p*-value < 0.01, Dunnett T3 multiple comparisons tests following one-way ANOVA), restoring sensitivity to TMZ (Fig. 4A, Supplemental Table S5). Indeed, LN-340 cells treated with O6BG in combination with TMZ showed a robust increase in the rate of DSBs as compared to TMZ treatment alone. This is in line with the MGMT-mediated resistance in this cell line. In contrast, when treating the cells in combination with JQ1, a significant difference (adjusted *p* < 0.01) of γ-H2AX levels was observed upon TMZ treatment, as compared to TMZ alone, indicating that depletion of MGMT upon JQ1 treatment led to an increase in DSB formation following TMZ treatment (Fig. 4A). However, no interaction was observed between O6BG and JQ1, hence the addition of O6BG in JQ1 treated cells did not further sensitize cells to TMZ, suggesting that MGMT protein levels were already low from JQ1 treatment.

To further support our findings of a MGMT-dependent effect of BETi and to confirm the key role of MGMT in conferring resistance to TMZ in these GBM models, we used a Dox-inducible Tet-On system for *MGMT* in the GBM line LN-229. LN-229 does not express endogenous *MGMT*, due to promoter methylation [19], and is known to be highly sensitive to TMZ treatment [33–35]. Induction of *MGMT* with Dox at 100 [ng/ml] yielded MGMT protein levels comparable to MGMT-proficient cell lines (Supplemental Fig. S6). We observed that LN-229MGMT^ind^_C12 cells acquired a strong TMZ resistance phenotype upon MGMT induction (Fig. 4B, Supplemental Table S5). Expectedly, the use of the pharmacologic MGMT inhibitor (MGMTi) O6BG had a significant effect on γ-H2AX levels (*p* < 0.01), restoring TMZ sensitivity, reflected by increased DSBs. In contrast, no effect (adjusted *p* > 0.5) was observed with JQ1 treatment, hence, not sensitizing Dox-treated LN-229MGMT^ind^_C12 cells to TMZ treatment in this context. This suggested that JQ1 was not able to interfere with ectopic *MGMT* expression, which is controlled by the Dox-inducible Tet-On promoter. Therefore, BETi did not influence ectopic MGMT expression or sensitivity to TMZ induced DSBs, whereas pharmacologic depletion of MGMT by O6BG treatment reversed the acquired TMZ resistance.

Altogether, our data have shown that JQ1 induces more DNA DSBs in TMZ treated GBM cells expressing endogenous *MGMT* as compared to TMZ alone.

### BET inhibition attenuates glioblastoma viability upon TMZ treatment

**Fig. 5:**
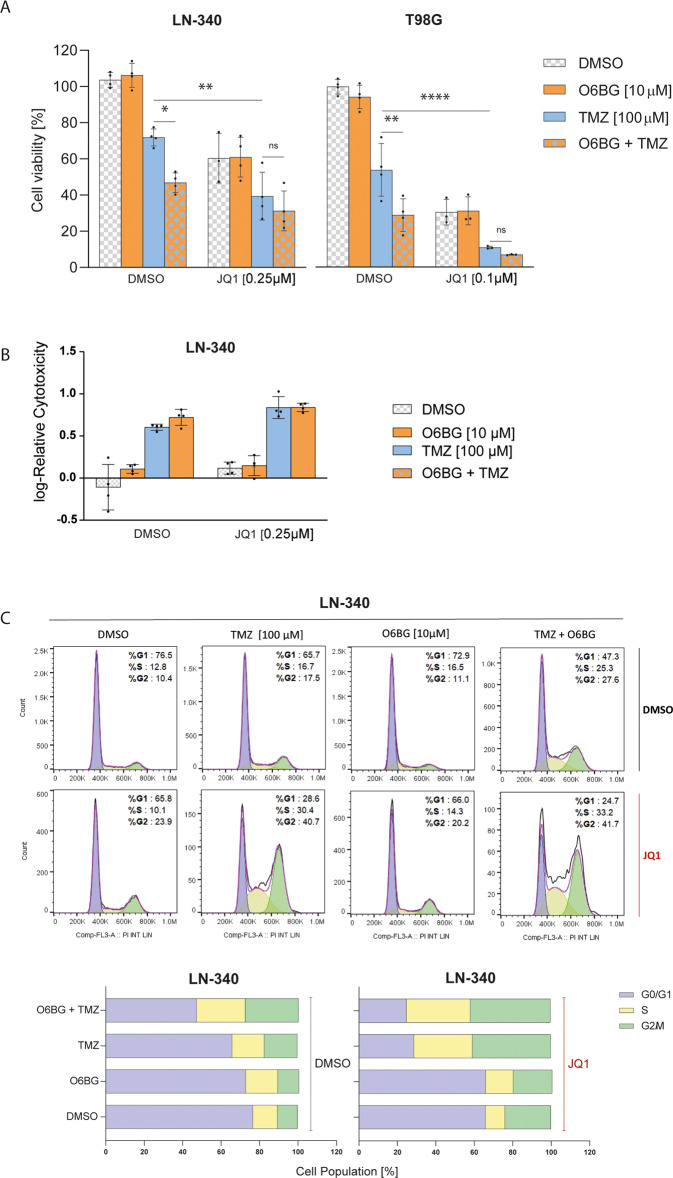
BETi sensitizes GBM to TMZ. **A** Cell viability was assessed in LN-340, and T98G. Cells were treated with JQ1 for 5 days at [0.25 μM] and [0.100 μM], respectively. On day 5, cells were treated with O6BG [10 µM] alone, or together with TMZ [100 µM]. Two additional TMZ treatments were given every 6 h, for a total of 3 TMZ treatments on day 5. End-point was set at 96 h after TMZ treatments. Data represent mean of 4 biological replicates. Adjusted *p-*values were determined by Tukey’s multiple comparisons tests following additive mixed model including interaction between the treatments (TMZ, OB6G and JQ1). Error bars are SD. *(*p* ≤ 0.05), **(*p* ≤ 0.01), ****(*p* < *P* ≤ 0.0001). **B** Relative cell death (Cytotox Red / cell confluency; Incucyte Zoom) was evaluated using the treatment conditions described in (**A**) monitored by live imaging over 70 h post treatment, comparison at 70 h. The mean of 4 biological replicates is shown. For stabilization and normal distribution of the data, log10-transformation was performed (Spearman correlation: -0.2423). **C** Analysis of the effect of JQ1 on the cell cycle profile of TMZ treated LN-340 cells was performed by FACS analysis using DAPI staining and subsequent flow cytometry cell cycle analysis using FlowJo. Cells were treated as in (**A**) and analyzed 48 h after TMZ treatment. Data represent 1 representative biological replicate.

The observed increase of DSBs suggested that treatment with JQ1 may reduce the viability of GBM cell lines with endogenous *MGMT* expression, in response to TMZ treatment. We treated LN-340 and T98G with JQ1 or TMZ alone or combined with JQ1, while both single agent treatments had a significant effect on cell viability in both cell lines (for both lines and both treatments, *p* < 0.0001, fixed effect from mixed model with interactions between treatments, Supplemental Table S6), we observed that the addition of JQ1 significantly sensitized cells to TMZ treatment (Fig. 5A), reflected in the significant interaction effect between JQ1 and TMZ (*P* = 0.006 and *p* < 0.0001, respectively). The specificity of the MGMT-mediated effect of JQ1-treatment was further tested using the pharmacologic inhibitor O6BG in the experiments, with or without JQ1, respectively. The addition of O6BG on its own had no effect on cell viability, whereas it sensitized the cells in combination with TMZ. However, O6BG did not further sensitize the cells to TMZ in presence of JQ1 (no significant interaction between O6BG and JQ1, *p* = 0.2746). A similar pattern was observed determining relative cell death 70 h after the treatment with TMZ by live imaging (IncuCyte Cytotox Red), although statistical significance was not reached (Fig. 5B). Cell proliferation (confluence, measured as % coverage by phase contrast) and cell death (area of fluorescence, red channel, μm^2^/image) is shown over the time course of 70 h in Supplemental Fig. S7.

Cell cycle analysis revealed that TMZ or JQ1 treatment alone did not alter the cell cycle profile compared to untreated cells. However, combinatorial treatment of JQ1 and TMZ increased S phase and G2/M phase cell cycle arrest in GBM cells as compared to controls (Fig. 5C). The addition of O6BG alone, or in combination with JQ1 had no effect on the cell cycle, while the combination of O6BG with TMZ increased the proportion of cells in S and G2/M phase (Fig. 5C).

### BET protein inhibition does not compromise the MMR system in glioblastoma

**Fig. 6:**
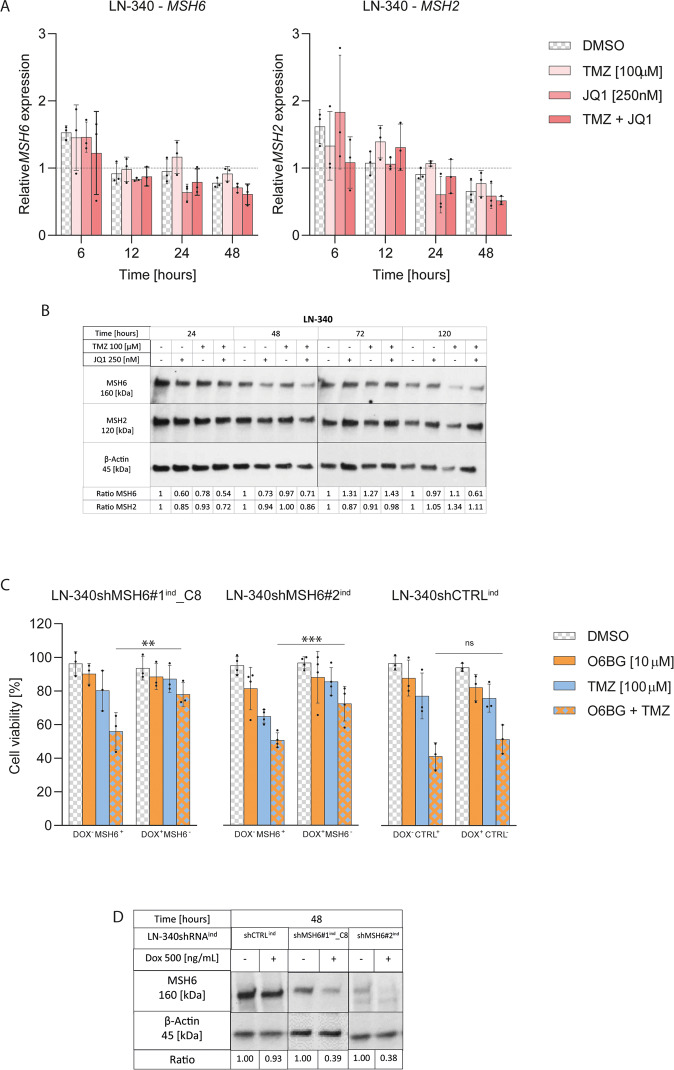
BETi does not impair the MMR pathway in GBM. **A** qRT-PCR analysis of relative *MSH6* and *MSH2* gene expression was performed in LN-340 at 6, 12, 24 and 48 h after treatment as indicated. Each data point represents an independent biological replicate. Data were normalized to the respective DMSO treatment for each time point (baseline). Adjusted *p*-values (*p*) were determined by Dunnett’s multiple comparisons test following two-way ANOVA using Geisser-Greenhouse correction for variance heterogeneity and including interaction between time and treatment. Error bars are SD. **B** Protein expression analysis of MSH6, MSH2 and β-Actin by WB at 24, 48, 72 and 120 h after treatment. One of 3 biological replicates is shown (corresponds to the experiment shown in Fig. 2B, same β-Actin control). Quantification of the replicates is available in Supplemental Fig. S8A. **C** Cell viability was performed on LN-340shMSH6#1^ind^_C8, LN-340shMSH6#2^ind^ and LN-340shCTRL^ind^. Cells were treated with Dox [500 ng/ml] for 5 days to induce expression of the respective shRNAs. On day 5, cells were treated with O6BG [10 µM] together with TMZ [100 µM]. 2 additional TMZ treatments were given every 6 h, for a total of 3 TMZ treatments on day 5. End-point was set at 96 h after TMZ treatments. Data points represent independent biological replicates. Adjusted *p*-values were determined by Tukey’s multiple comparisons tests following three-way ANOVA including interaction between the treatments (TMZ, OB6G and JQ1). **D** WB, knockdown validation for the dox-inducible shRNAs systems. Error bars are SD. *(*p* ≤ 0.05), **(*p* ≤ 0.01), ***(*P* ≤ 0.001). Full length western blots are available in Supplemental Material.

As aforementioned, a compromised MMR system generates resistance to TMZ treatment, as it is essential for the cytotoxic effect O6meG lesions that remain unrepaired in absence of MGMT. Our differential gene expression analysis in LN-2683GS had shown that key MMR genes, were only transiently modulated by JQ1 treatment and their expression was restored after 12 h (Fig. 1). Treatment with JQ1 alone or in combination with TMZ did not significantly alter RNA or protein expression levels of *MSH6* and *MSH2* in LN-340 (Fig. 6A, B, Supplemental Table S8), T98G or the sphere line MDA-GSC-23 (Supplemental Fig. S8).

To determine the effect of a non-functional MMR pathway in conferring TMZ resistance in our experimental model, we transduced LN-340 with a Dox-inducible Tet-On shRNA against *MSH6* using 2 distinct sequences (Fig. 6D). Depletion of *MSH6* confirmed that TMZ resistance was independent of MGMT in this scenario, as pharmacologic inhibition of endogenous MGMT with O6BG was ineffective in restoring sensitivity to TMZ as measured by cell viability (adjusted *p*-values = 0.0022 and 0.0002 for the 2 sequences, three-way ANOVA including interaction between the treatments, TMZ, OB6G and JQ1, corrected for multiple testing by Tukey’s test). On the contrary, cells transduced with the non-targeting Dox-inducible Tet-On shRNA did not change behavior upon doxycycline exposure and remained sensitized to TMZ upon O6BG treatment (Fig. 6C, Supplemental Table S9) (adjusted *p*-value = 0.127).

Finally, we demonstrated that the use of BETi in GBM cells does not negatively impact the MMR system that would result in undesirable TMZ resistance.

## Discussion

Changes of the epigenetic landscape in tumors contribute to all hallmarks of cancer and have been recognized as promising targets for treatment. Encouraging preclinical results have been obtained with small molecule inhibitors targeting BET proteins that are epigenetic modifiers and have been associated with overexpression of cancer relevant pathways [5]. While some single agent efficacy has been observed in preclinical GBM models [36–38] the challenge is to find specific synergistic combination therapies [6, 7, 39, 40]. In the present study we aimed at identifying rational combination therapies by leveraging potential vulnerabilities emerging upon disturbing GBM cells with BETi in the context of DDR. The significantly modulated genes displayed six main gene expression response patterns to JQ1 treatment. This revealed *MGMT* as a consistently downregulated gene, rendering it a top candidate, due to its pivotal clinical relevance of conferring resistance to TMZ in GBM patients. This finding provides a potential novel therapeutic strategy to inhibit *MGMT* expression and sensitize patients with an unmethylated *MGMT* promoter to TMZ therapy, who normally have no benefit from such treatment [10].

Indeed, by combining BETi with TMZ, we demonstrated enhanced DNA DSB levels and reduced cell viability compared to single agent TMZ in GBM cells expressing endogenous MGMT. However, in light of the large number of the BETi-disturbed genes that contribute to the efficacy of the drug as anticancer agent through other mechanisms [41, 42], we sought to demonstrate the specificity of BETi to downregulate *MGMT* expression and its potential to prohibit MGMT induction upon TMZ treatment. We observed reduction of BRD4 coverage at the promoter of *MGMT*, in concordance with a reduction of Pol II binding, and the associated decrease in *MGMT* expression levels. Moreover, ectopic expression of *MGMT* from an artificial promoter could not be attenuated by JQ1, while pharmacologic MGMT depletion restored sensitivity to TMZ. The MGMT-specific effect under TMZ treatment is further supported by the fact that combination of BETi with pharmacologic inhibition of MGMT did not further decrease cell viability or increase cell death. These findings are also reflected in the alteration of the cell cycle profile of MGMT-positive cells that showed an increased cell population in S and G2/M phase, when the TMZ treatment was combined with the specific MGMT inhibitor O6BG or JQ1 that both deplete MGMT. Interestingly, we noticed that the magnitude of the TMZ effect was much greater in JQ1 treated cells compared to cells pre-treated with O6BG followed by TMZ only. This suggests that other BETi-related changes may interact with the modulation of the cell cycle in addition to, or synergistic with the effect of TMZ in MGMT-deficient (depleted or silenced) cells. Taken together, this provides supportive evidence for a causal relationship of BETi in the depletion of *MGMT* and emphasizes the specificity of BETi to sensitize MGMT-proficient cells to TMZ treatment.

At the same time we provided evidence that the MMR pathway is not affected by BETi which is highly relevant, as it would induce unwanted resistance to alkylating agents even in absence of MGMT. Inactivation of MMR is an important resistance mechanism rendering treatment with alkylating agents ineffective [43, 44]. Therefore, it is of note that the BETi response pattern of the MMR pathway exhibited only transient downregulation, which we confirmed in several cell lines, exemplified for the key members of MMR, *MSH6* and *MSH2*. The sensitivity of our in vitro model to attenuate MMR, was illustrated by depleting MSH6 from the cells. Resistance to TMZ was observed, even upon pharmacologic inactivation of MGMT as expected, and reported by others [13, 27].

Small-molecule BET inhibitors have similar features in in vitro models, allowing for mechanistic evaluations of the mode of action using a tool drug, like JQ1 as we described in this study. We have previously reported that BETi-specific responses can be measured in orthotopic GBM xenografts measuring BETi-responsive gene signatures of interest, including the pharmacologic marker for target engagement of BETi, *HEXIM1* that is rapidly upregulated [6]. This allows to functionally evaluate whether the drug concentration in the tumor reaches sufficiently high levels to induce the desired biological effect, and second, informs on the activity of the drug on the hypothesized mechanism in the target tissue.

Major concerns for combination therapies are overlapping toxicity. Previous attempts to specifically target MGMT comprised depletion of MGMT using pharmacologic inhibitors, such as pseudosubstrates like O6BG or its derivative PaTrin-2 [45]. However, they have failed in the clinic due to overlapping toxicity with TMZ and other alkylating agents [46, 47]. Hence, the potential of the synergistic effect between the BETi-MGMT-mediated efficacy and cytotoxicity conferred through modulation of other, MGMT-unrelated cancer relevant pathways will be of importance for successful therapy without overt toxicity.

Clinical trials are currently ongoing for testing BETi in GBM patients. Encouraging results have been reported recently from a phase 1b trial in newly diagnosed GBM, suggesting good tolerability of the BETi CC-90010 in combination with radio-chemotherapy with TMZ, the current standard of care [48]. An ongoing window of opportunity study with the same drug in recurrent and progressive glioma and GBM (clinicaltrials.gov, NCT04047303) could be leveraged to gain information, not only to test the penetration of the BBB by the drug, but also to provide functional evidence for the efficiency of BETi-mediated *MGMT* depletion (RNA and sustained depletion of protein) and of other candidates of interest. The investigation of BETi response signatures will provide a useful tool to gain insights for the design of future clinical studies investigating novel combination therapies.

Our study has provided novel mechanistic evidence for a causal relationship between BETi and MGMT depletion, and the property to sensitize GBM cells to TMZ. This is in support of rational combination of BETi with TMZ in GBM, in particular promising for benefiting patients with MGMT unmethylated GBM.

## Supplementary information

Supplementary Tables & Figures

Supplementary Table S3

Original Data File

check list

## Data Availability

Due to patient privacy protection, the raw sequencing data will be made available upon request. Methylome data (EPIC BeadChip) for MDA-GSC-23 are available at the Gene Expression Omnibus database (GEO, http://www.ncbi.nlm.nih.gov/geo/) under the accession number GSE217515.
