# Supplementary material for: BET protein inhibition sensitizes glioblastoma cells to temozolomide treatment by attenuating MGMT expression
Source: Cell Death Dis. 2022 Dec 13;13(12):1037. doi: 10.1038/s41419-022-05497-y (PMC9747918; doi:10.1038/s41419-022-05497-y)
Supplement: Supplementary file 1 — Supplementary Tables & Figures [file 41419_2022_5497_MOESM1_ESM.pdf]

Supplementary Tables and Figures

Content:

Supplementary Table S1. MGMT promoter methylation status cell lines & sphere lines

Supplementary Table S2. Primer sequences.

Supplementary Table S3. Description of the 169 DDR genes affected by JQ1 treatment.

Description: The table contains gene expression levels, DDR annotation and classification of the genes into the six JQ1-treatment-related response profiles. See separate excel file.

Supplementary Table S4. Statistical details of comparisons in Figure 2

Supplementary Table S5. Statistical details of comparisons in Figure 4

Supplementary Table S6. Statistical details of comparisons in Figure 5A

Supplementary Table S7. Statistical details of comparisons in Figure 5B

Supplementary Table S8. Statistical details of comparisons in Figure 6A

Supplementary Table S8. Statistical details of comparisons in Figure 6C

Supplementary Figures S1-S8

References

Supplementary Table S1.

A *MGMT* promoter methylation status of cell lines & sphere lines

Cell lines & Sphere lines	**MGMT* methylation status	Reference
LN-18	U	Sciuscio *et al* 2011
LN-229	M	Sciuscio *et al* 2011
LN-340	U/M	
LN-382	U/M	
T98G	U/M	
LN-2683GS	U/M	Sciuscio *et al* 2011
LN-4372GS	U/M	
MDA-GSC-23 (GSC23, RRID:CVCL_DR59**)**	U/M	

*Status determined by Methylation Specific PCR (MSP) [1, 2]
Abbreviations: U, unmethylated; M, methylated; U/M, unmethylated and methylated
Sciuscio et al. 2011 [3]

B STR Finger print of LN-4372GS and corresponding PBMC

Marker	LN-4372GS	TuBa 4372 PBMCs
**Amelogenin**	XY	XY
**D3S1358**	15 - 18	15 - 18
**D1S1656**	13 - 15.3	13 - 15.3
**D2S441**	11	11
**D10S1248**	15	15
**D13S317**	14	8 - 14
**Penta_E**	7 - 12	7 - 12
**D16S539**	11 - 13	11 - 13
**D18S51**	13 - 17	13 - 17
**D2S1338**	20 - 25	20 - 25
**CSF1PO**	11 - 12	11 - 12
**Penta_D**	11 - 12	11 - 12
**TH01**	7 - 8	7 - 8
**vWA**	15 - 17	15 - 17
**D21S11**	28 - 30	28 - 30
**D7S820**	9 - 11	9 - 11
**D5S818**	9 - 11	9 - 11
**TPOX**	8 - 9	8 - 9
**D8S1179**	15	15
**D12S391**	19 - 22	19 - 22
**D19S433**	13 - 14	13 - 14
**SE33**	20.2 - 31.2	20.2 - 31.2
**D22S1045**	16	16 - 17
**FGA**	21 - 23	21 - 23

Supplementary Table S2

A qPCR primers

Gene	Forward primer (5'-3')	Reverse primer (5'-3')
GAPDH	AGGTGAAGGTCGGAGTCAACG	CGTTCTCAGCCTTGACGGTG
HEXIM1	AAGGACTAGCTAAAGGCGTCAC	TGGCTAGTAGAGTCCTCGAAGTTT
MGMT	GCTGCGGTTCTCGGAGGTC	CTGCCAGGGCTGCTAATTGC
MSH6	CACCAGGAGATTTGGTTTGG	TGTTGGGCTGTCATCAAAAA
MSH2	GACCGGGGCGACTTCTATAC	GCCCCATGTACTTGATCACC

B ChIP-qPCR primers

Gene	Forward primer (5'-3')	Reverse primer (5'-3')
MGMT_F2	AAAAGGTACGGGCCATTTG	CAGTCTGCGCATCCTCG
MGMT_F3	GCGCTCTCTTGCTTTTCTCA	GACACTCACCAAGTCGCAAA
H19	Undisclosed – Diagenode Kit	Undisclosed – Diagenode Kit
Myoglobin exon 2	Undisclosed – Diagenode Kit	Undisclosed – Diagenode Kit

Supplementary Table S4: Supplementary Statistics to Figure 2A

Cell lines	ANOVA table	SS	DF	MS	F (DFn, DFd)	P value
**LN-340**	Time x Trt	0.1529	6	0.02548	F (6, 18) = 0.5471	P=0.7658
	Time	0.9419	3	0.314	F (1.738, 10.43) = 6.742	P=0.0153
	Trt	1.097	2	0.5487	F (2, 6) = 11.56	P=0.0087
	Subject	0.2847	6	0.04745	F (6, 18) = 1.019	P=0.4443
	Residual	0.8383	18	0.04657		
**T98G**	Time x Trt	0.2745	6	0.04575	F (6, 18) = 1.293	P=0.3098
	Time	0.1817	3	0.06058	F (1.829, 10.97) = 1.712	P=0.2254
	Trt	0.5377	2	0.2689	F (2, 6) = 5.300	P=0.0472
	Subject	0.3044	6	0.05073	F (6, 18) = 1.433	P=0.2562
	Residual	0.637	18	0.03539		
**LN-2683GS**	Time x Trt	0.0682	6	0.01137	F (6, 18) = 1.860	P=0.1436
	Time	0.5984	3	0.1995	F (1.529, 9.174) = 32.64	P=0.0001
	Trt	2.59	2	1.295	F (2, 6) = 330.2	P<0.0001
	Subject	0.02354	6	0.003923	F (6, 18) = 0.6418	P=0.6958
	Residual	0.11	18	0.006112		
**LN-4372GS**	Time x Trt	0.1366	6	0.02276	F (6, 9) = 0.2850	P=0.9298
	Time	0.22	3	0.07333	F (1.491, 4.473) = 0.9180	P=0.4324
	Trt	2.079	2	1.039	F (2, 3) = 46.43	P=0.0055
	Subject	0.06716	3	0.02239	F (3, 9) = 0.2802	P=0.8384
	Residual	0.7189	9	0.07988		

Supplementary Table S5: Supplementary Statistics to Figure 4

Cell lines	ANOVA table	SS	DF	MS	F (DFn, DFd)	P value
LN-340	Treatment	1798496	7	256928	F (7, 16) = 16.53	P<0.0001
	Residual	248636	16	15540		
	Total	2047132	23			
LN-229MGMT+C12_NO DOX	Treatment	1537781	7	219683	F (7, 16) = 5.780	P=0.0018
	Residual	608072	16	38004		
	Total	2145853	23			
LN-229MGMT+C12_DOX	Treatment	1065768	7	152253	F (7, 16) = 7.332	P=0.0005
	Residual	332242	16	20765		
	Total	1398010	23			

Supplementary Table S6: Supplementary Statistics to Figure 5A

Cell lines	Fixed effects (type III)	P value	F (DFn, DFd)
LN-340	JQ1	<0.0001	F (1, 23) = 104.4
	O6BG	0.0347	F (1, 23) = 5.038
	TMZ	<0.0001	F (1, 23) = 112.8
	JQ1 x O6BG	0.2746	F (1, 23) = 1.253
	JQ1 x TMZ	0.006	F (1, 23) = 9.165
	O6BG x TMZ	0.0123	F (1, 23) = 7.375
	JQ1 x O6BG x TMZ	0.1736	F (1, 23) = 1.972
T98G	JQ1	<0.0001	F (1, 8) = 561.8
	O6BG	0.0523	F (1, 12) = 4.637
	TMZ	<0.0001	F (1, 12) = 104.3
	JQ1 x O6BG	0.0122	F (1, 8) = 10.40
	JQ1 x TMZ	<0.0001	F (1, 8) = 55.99
	O6BG x TMZ	0.1735	F (1, 12) = 2.094
	JQ1 x O6BG x TMZ	0.0969	F (1, 8) = 3.535

Supplementary Table S7: Supplementary Statistics to Figure 5B

ANOVA table	SS	DF	MS	F (DFn, DFd)	P value
JQ1	0.1918	1	0.1918	F (1, 24) = 12.35	P=0.0018
TMZ	3.743	1	3.743	F (1, 24) = 241.0	P<0.0001
O6BG	0.06672	1	0.06672	F (1, 24) = 4.295	P=0.0491
JQ1 x TMZ	0.003787	1	0.003787	F (1, 24) = 0.2438	P=0.6260
JQ1 x O6BG	0.04604	1	0.04604	F (1, 24) = 2.964	P=0.0980
TMZ x O6BG	0.007965	1	0.007965	F (1, 24) = 0.5128	P=0.4808
JQ1 x TMZ x O6BG	0.002526	1	0.002526	F (1, 24) = 0.1626	P=0.6903
Residual	0.3728	24	0.01553		

Supplementary Table S8: Supplementary Statistics to Figure 6A

Cell line	ANOVA table	SS	DF	MS	F (DFn, DFd)	P value
**LN-340_MSH6**	Time x Trt	0.3011	9	0.03346	F (9, 24) = 0.5867	P=0.7950
	Time	3.081	3	1.027	F (1.363, 10.91) = 18.01	P=0.0008
	Trt	0.5032	3	0.1677	F (3, 8) = 2.488	P=0.1347
	Subject	0.5395	8	0.06743	F (8, 24) = 1.182	P=0.3497
	Residual	1.369	24	0.05703		
**LN-340_MSH2**	Time x Trt	1.415	9	0.1573	F (9, 24) = 1.676	P=0.1502
	Time	4.888	3	1.629	F (1.784, 14.27) = 17.36	P=0.0002
	Trt	0.2405	3	0.08015	F (3, 8) = 0.6038	P=0.6307
	Subject	1.062	8	0.1327	F (8, 24) = 1.414	P=0.2410
	Residual	2.253	24	0.09386		

Supplementary Table S9: Supplementary Statistics to Figure 6C

cell lines	ANOVA table	SS	DF	MS	F (DFn, DFd)	P value
LN-340shMSH6#1	JQ1	224.8	1	224.8	F (1, 8) = 10.70	P=0.0113
	O6BG	749.2	1	749.2	F (1, 8) = 6.047	P=0.0394
	TMZ	1689	1	1689	F (1, 8) = 13.63	P=0.0061
	JQ1 x O6BG	96.65	1	96.65	F (1, 8) = 4.599	P=0.0643
	JQ1 x TMZ	415.3	1	415.3	F (1, 8) = 19.76	P=0.0022
	O6BG x TMZ	187.8	1	187.8	F (1, 8) = 1.515	P=0.2533
	JQ1 x O6BG x TMZ	72.72	1	72.72	F (1, 8) = 3.460	P=0.0999
	Subject	991.2	8	123.9		
	Residual	168.1	8	21.01		
LN-340shMSH6#2	JQ1	1288	1	1288	F (1, 12) = 58.65	P<0.0001
	O6BG	1231	1	1231	F (1, 12) = 8.776	P=0.0119
	TMZ	3872	1	3872	F (1, 12) = 27.60	P=0.0002
	JQ1 x O6BG	19.25	1	19.25	F (1, 12) = 0.8765	P=0.3676
	JQ1 x TMZ	586.8	1	586.8	F (1, 12) = 26.72	P=0.0002
	O6BG x TMZ	12.48	1	12.48	F (1, 12) = 0.08897	P=0.7706
	JQ1 x O6BG x TMZ	8.226	1	8.226	F (1, 12) = 0.3746	P=0.5519
	Subject	1683	12	140.3		
	Residual	263.5	12	21.96		
LN-340shCTRL	JQ1	0.1452	1	0.1452	F (1, 8) = 0.003922	P=0.9516
	O6BG	2463	1	2463	F (1, 8) = 22.06	P=0.0015
	TMZ	4967	1	4967	F (1, 8) = 44.49	P=0.0002
	JQ1 x O6BG	26.14	1	26.14	F (1, 8) = 0.7060	P=0.4252
	JQ1 x TMZ	107.3	1	107.3	F (1, 8) = 2.899	P=0.1270
	O6BG x TMZ	588.7	1	588.7	F (1, 8) = 5.274	P=0.0508
	JQ1 x O6BG x TMZ	76.88	1	76.88	F (1, 8) = 2.076	P=0.1876
	Subject	893.1	8	111.6		
	Residual	296.2	8	37.03		

SUPPLEMENTARY FIGURES

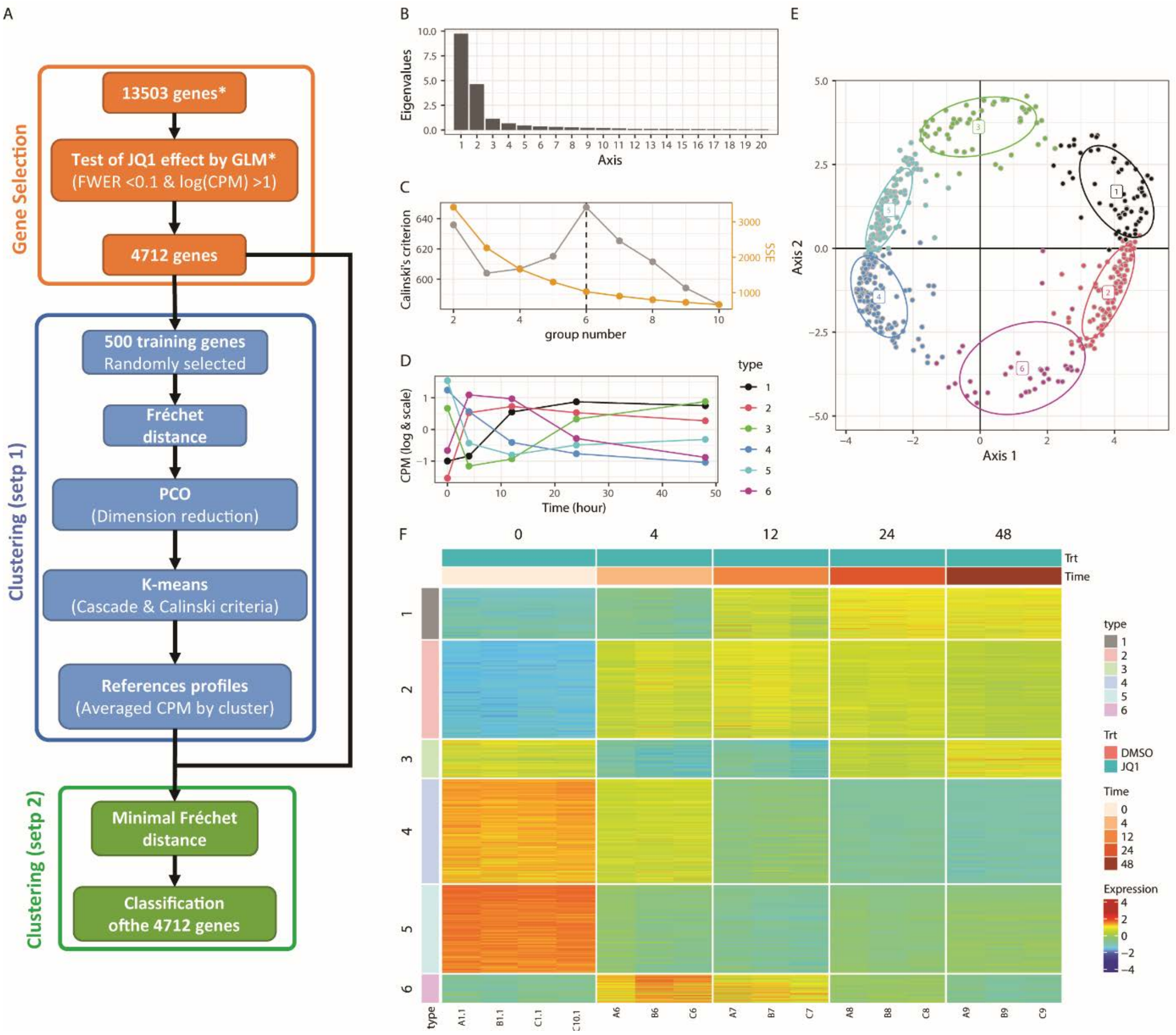

Supplementary Figure 1. Trajectory classification of the genes affected by JQ1-treatment. (A) Flow diagram related two the two-step classification method. The 4721 genes were initially selected by testing the interaction between time and JQ1-treatment in the GBM derived sphere line LN-2683GS [4]. The first step of the classification consisted in the construction of the 6 reference profiles of response to JQ1 treatment. In a second step, the group assignment for each gene was given by minimal Fréchet distance between gene profiles and reference profiles. (B) Eigenvalues from principal coordinates analysis (PCO) based on the Fréchet distance matrix of the 500 randomly selected genes. The three first components of the PCO were then used to partition the 500 selected genes. (C) The cluster (group) number from the cascade K-means analysis is represented in function of the *Calinski's criterion* [5] and SSE (sum of squared errors). The optimal group number is given by the maximal Calinski value (6 groups). (D) The 6 averaged expression patterns of the 500 randomly selected genes (reference profiles; CPM, counts per million) are displayed upon clustering over the time course of treatment. (E) representation of the 6 clusters on the first vectorial plane of the principal coordinates analysis (PCO) based on the 500 randomly selected genes. The heatmap (**F**) illustrates the normalized expression of the 4712 selected genes upon JQ1 treatment over a time course of 48h. The gene trajectories were classified in the 6 clusters of response to the treatment defined by the two-step procedure based on Fréchet distances.

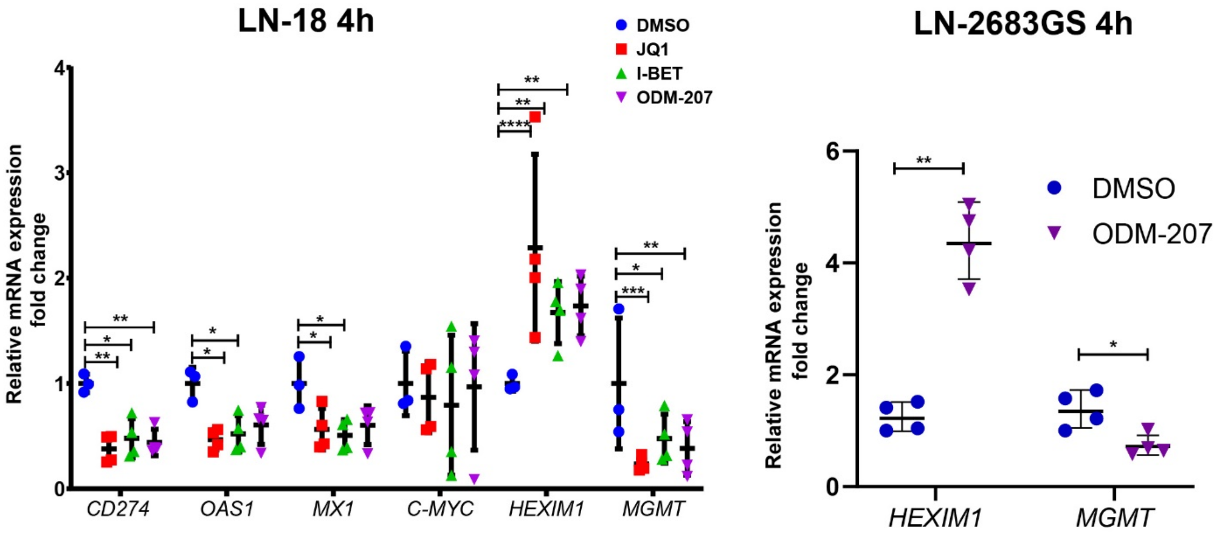

Supplementary Figure S2. BET-inhibitors affect gene expression similarly in *in vitro* experiments.

The GBM cell-line LN-18 with endogenous *MGMT* expression was treated for 4h with the BET-inhibitors JQ1, I-BET, or ODM-207. Expression was determined by qRT-PCR normalized to *GAPDH.* Expression was compared between the DMSO control and the respective BET-inhibitors. Data represent a summary of 3-4 independent experiments. Error bars are SD. The data is normalized to the median of DMSO treated samples. P, individual P-value (not corrected for multiple testing, exact Fisher LSD test); P > 0.05, * P ≤ 0.05, **P ≤ 0.01, *** P ≤ 0.001, **** P ≤ 0.0001. Primers have been reported in Gusyatiner *et al* 2021 [4], or in Supplemental Table S1. *HEXIM1* expression is considered a pharmacodynamic marker for target engagement and activity of BET inhibitors [6]. The sphere line LN-2683GS, with endogenous *MGMT* expression, was treated for 4h with DMSO or ODM-207, analysis was performed as indicated above.

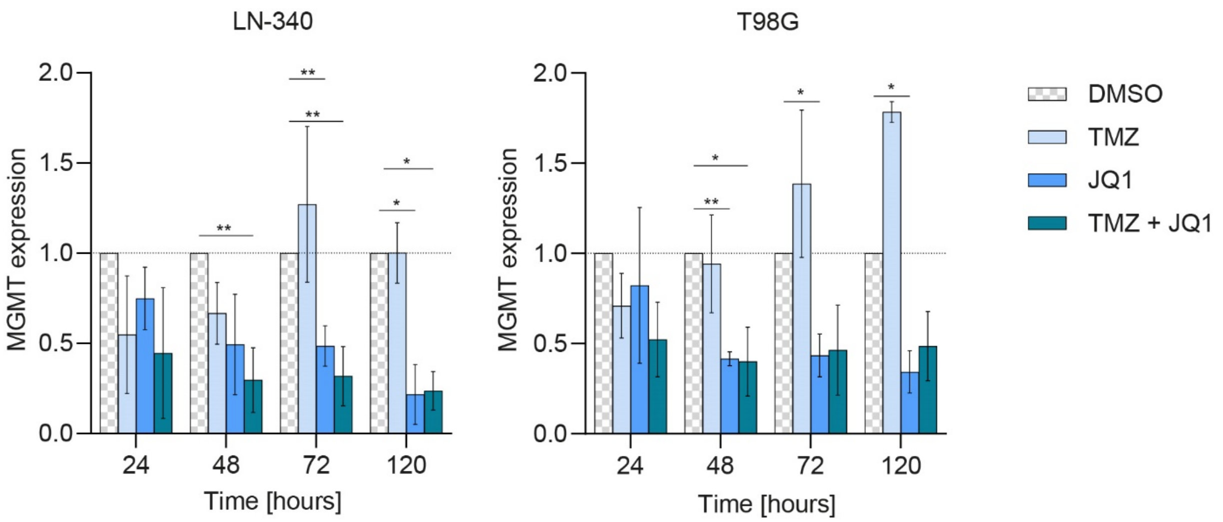

Supplementary Figure S3. Quantification of MGMT Western blots from Figure 2B.

The films of the western blots probed for MGMT and β–Actin were analyzed by densitometry. The ratio MGMT/ β–Actin of the DMSO controls were set to 1 and served as normalizer by time point. The quantification is based on 3 biological replicates. Error bars are SD. P-values were obtained by two-way ANOVA, * (p≤0.05), ** (P≤ 0.01).

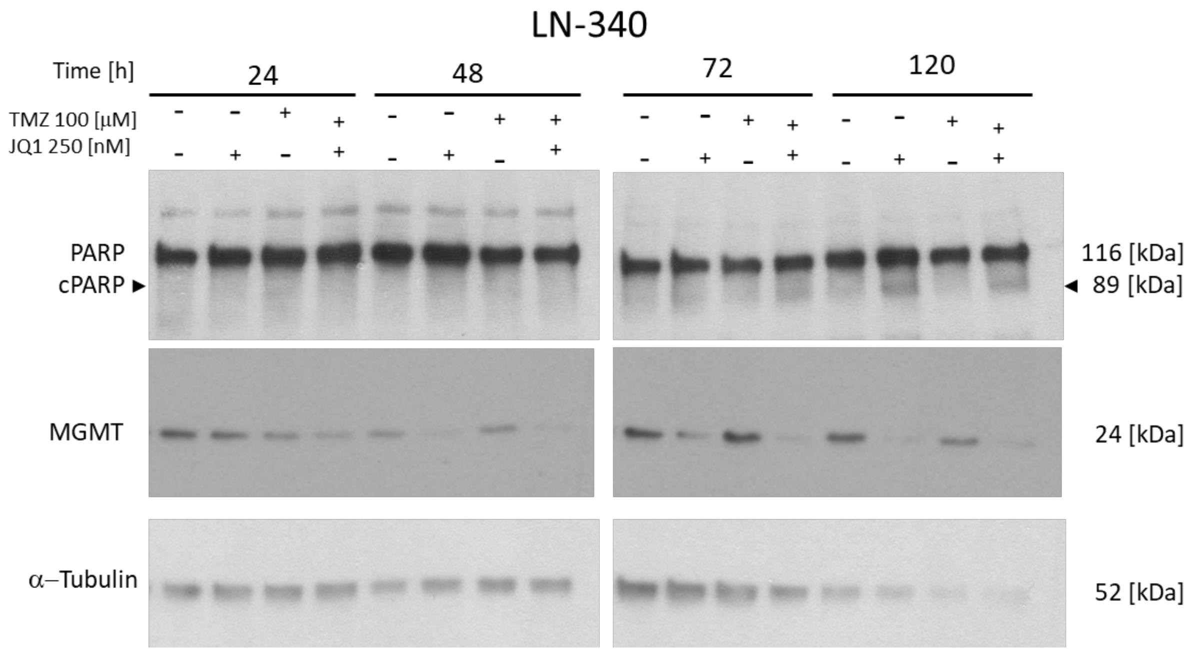

Supplementary Figure S4. JQ1 treatment alone induces PARP cleavage.

The experimental set-up corresponds to Figure 2B. Treatment of LN-340 with JQ1 at 250 nM JQ1 indicates PARP cleavage after 72 h and 120 h as indicated by Western. Of note, the TMZ treatment that in this experiment was administered at time 0, showed no effect on its own. This is in line with the fact that MGMT protein was present at this time point, known to rapidly repair TMZ-induced lesions. The short half-life of TMZ (1.8 h) at physiological pH precludes a TMZ effect at later time points, when MGMT is depleted by JQ1 in this experiment.

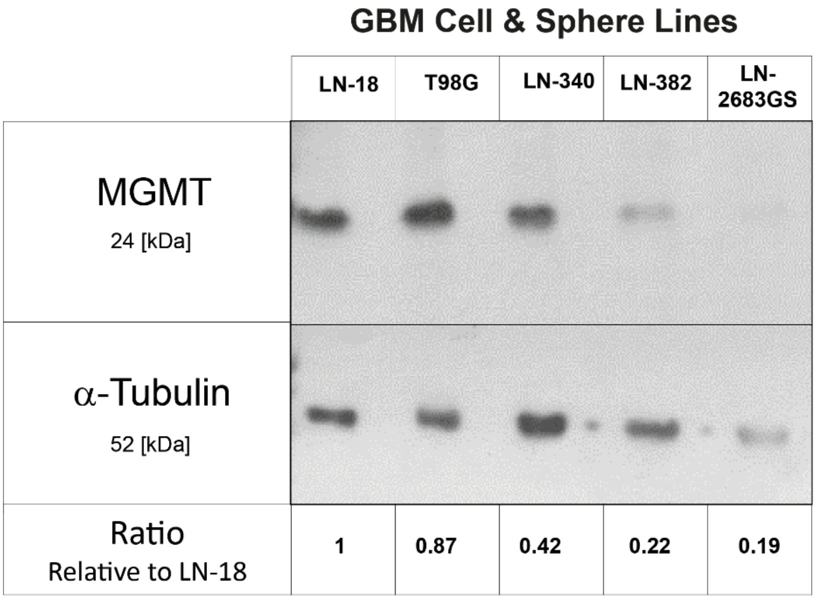

Supplementary Figure S5. **Basal MGMT expression in GBM cell and sphere lines**. MGMT protein expression normalized to LN-18 (unmethylated *MGMT* promoter).

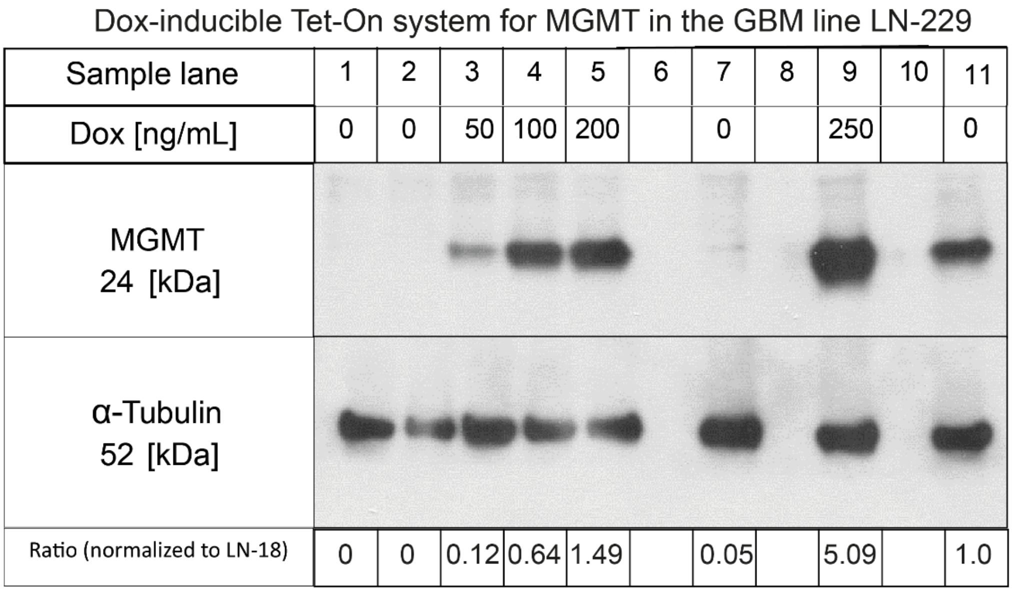

Supplemetary Figure S6. **Dox-Inducible MGMT in LN-229**.
The GBM cell line LN-229 transduced with a Dox-inducible *MGMT* construct was treated 48h with various concentrations of Dox (0-250ng/mL). MGMT protein expression was normalized to the endogenous MGMT levels of LN-18. 1 = LN-229 parental, 2 = LN-229MGMTind_C12 – untreated, 3 = LN-229MGMTind_C12 – Dox 50ng/mL, 4 = LN-229MGMTind_C12 – Dox 100ng/mL, 5 = LN-229MGMTind_C12 – Dox 200ng/mL, 6 = empty well, 7 = LN-229MGMTind_pool – untreated, 8 = empty well, 9 = LN-229MGMTind_pool – Dox 250ng/mL, 10 = empty well, 11 = LN-18 (MGMT positive control).

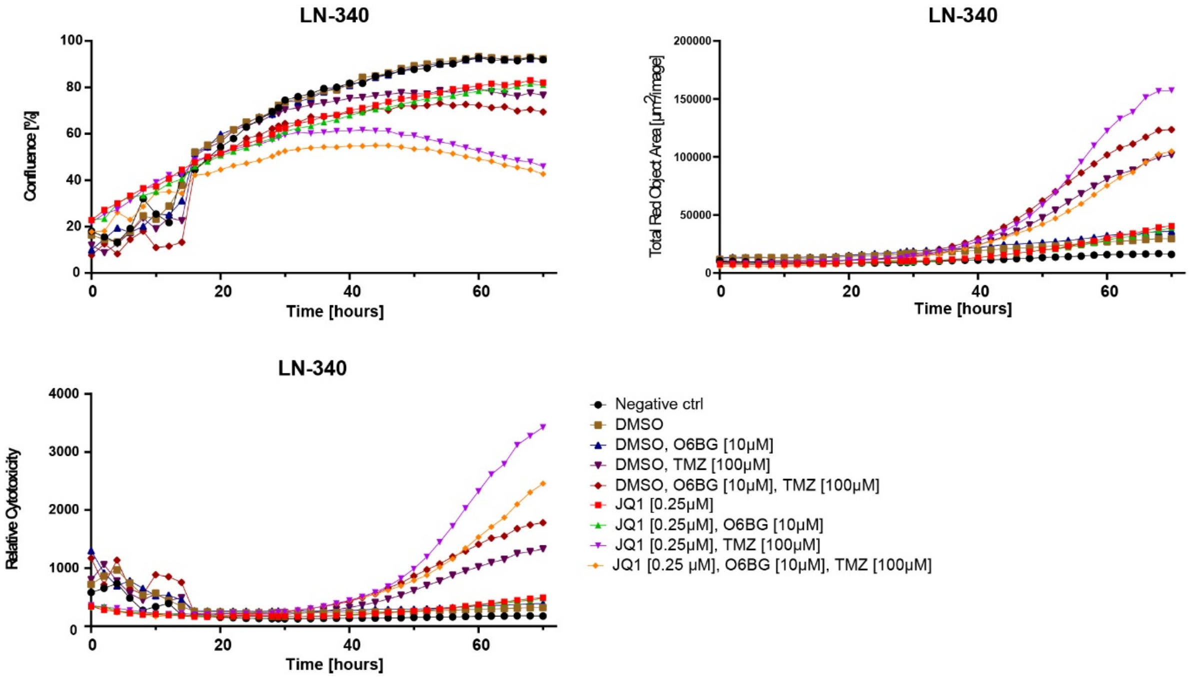

Supplementary Figure S7. **Relative cytotoxicity monitored by live imaging.**

Relative cytotoxicity was assessed in LN-340 by Incucyte Zoom imaging (Cytotox Red/ Confluence). Cells were treated with JQ1 for 5 days at [0.25µM]. On day 5, cells were treated with O6BG [10 µM] alone, or together with TMZ [100 µM]. Two additional TMZ treatments were given every 6h, for a total of 3 TMZ treatments on day 5. End-point was set at 70h after TMZ treatments. Cell confluence is measured as % coverage by phase contrast, and cell death, as area of Cytotx Red fluorescence in the red channel, µm^2/image. Data represent the mean of 3 technical replicates of one of 4 biological replicates quantified in Figure 5B.

A

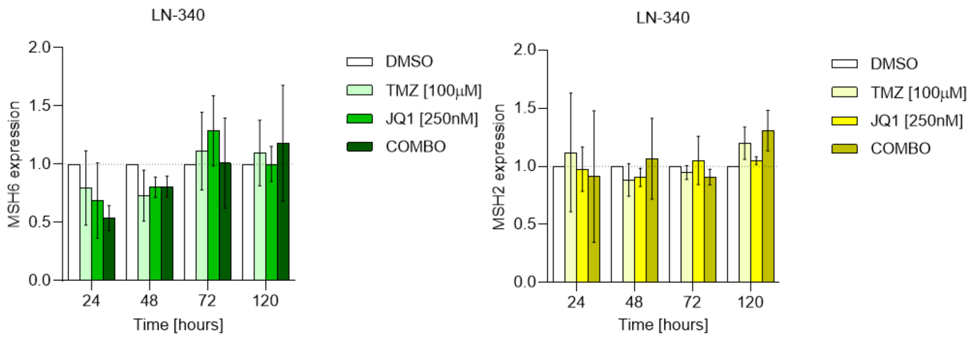

B

T98G

Time [hours]	24				48				72				120			
TMZ 100 [µM]	-	-	+	+	-	-	+	+	-	-	+	+	-	-	+	+
JQ1 100 [nM]	-	+	-	+	-	+	-	+	-	+	-	+	-	+	-	+

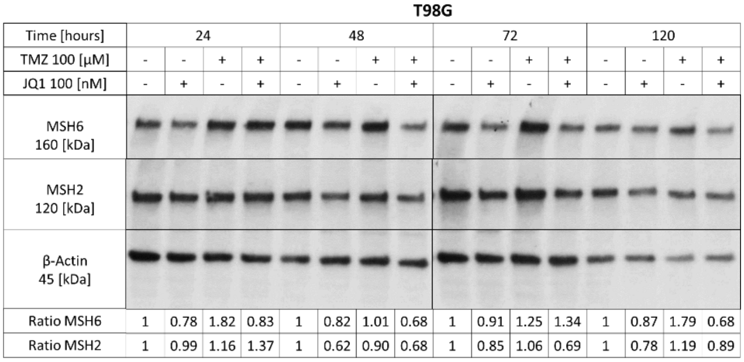

Ratio MSH6	1	0.78	1.82	0.83	1	0.82	1.01	0.68	1	0.91	1.25	1.34	1	0.87	1.79	0.68
Ratio MSH2	1	0.99	1.16	1.37	1	0.62	0.90	0.68	1	0.85	1.06	0.69	1	0.78	1.19	0.89

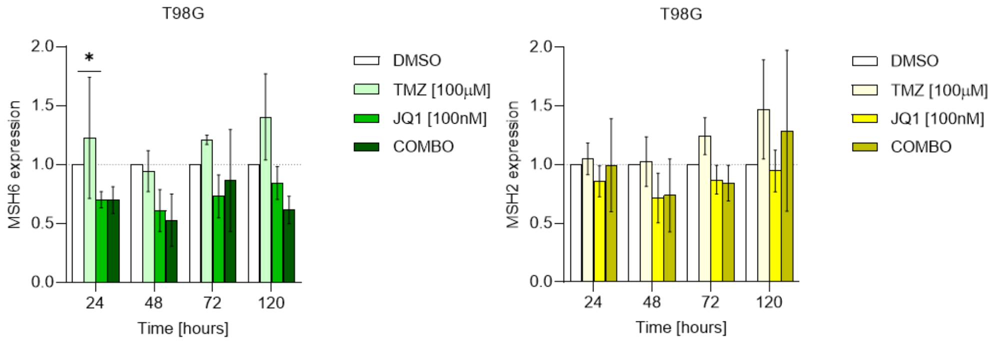

C

MDA-GSC-23

Time [hours]	24				48				72				120			
TMZ 100 [µM]	-	-	+	+	-	-	+	+	-	-	+	+	-	-	+	+
JQ1 250 [nM]	-	+	-	+	-	+	-	+	-	+	-	+	-	+	-	+

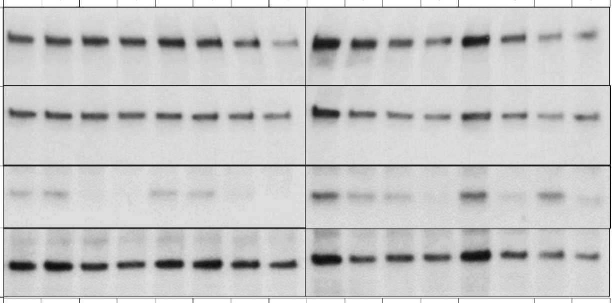

Ratio MSH6	1	0.93	1.36	1.32	1	0.77	0.78	0.54	1	1.35	0.78	0.79	1	1.1	0.80	1.07
Ratio MSH2	1	0.87	1.20	1.15	1	0.93	1.02	1.04	1	1.12	0.71	0.78	1	1.14	0.92	1.76
Ratio MGMT	1	1.22	0.58	0.36	1	0.86	0.52	0.40	1	0.80	0.61	0.29	1	0.38	1.50	0.69

Supplementary Figure S8. BETi does not impair the MMR pathway in GBM.

The effect of JQ1 on the expression of the MMR proteins MSH6 and MSH2 is shown for GBM cell and sphere lines over a time course of 24 to 120h, with or without treatment with TMZ (at time 0) as indicated. The films of the western blots, probed for the different proteins and β-

Actin, were analyzed by densitometry. The ratio of the proteins of interest /β-Actin of the DMSO controls were set to 1 and served as normalizer by time point. **A** Quantification of the Western blots from LN-340 based on 3 biological replicates associated to Figure 6B. **B** Representative Western blots are shown for T98G, and respective quantification based on 3 biological replicates. **C** Western blots are shown for the sphere line MDA-GSC-23^{luc} for MSH6, MSH2, and MGMT. The p-values were determined by two-way ANOVA. Error bars are SD, *($p \leq 0.5$).

References

1. Esteller M, Hamilton SR, Burger PC, Baylin SB, Herman JG. Inactivation of the DNA repair gene O6-methylguanine-DNA methyltransferase by promoter hypermethylation is a common event in primary human neoplasia. *Cancer Res* 1999, **59**(4)**:** 793-797.

2. Palmisano WA, Divine KK, Saccomanno G, Gilliland FD, Baylin SB, Herman JG*, et al.* Predicting lung cancer by detecting aberrant promoter methylation in sputum. *Cancer Research* 2000, **60**(21)**:** 5954-5958.

3. Sciuscio D, Diserens AC, van Dommelen K, Martinet D, Jones G, Janzer RC*, et al.* Extent and patterns of MGMT promoter methylation in glioblastoma- and respective glioblastoma-derived spheres. *Clin Cancer Res* 2011, **17**(2)**:** 255-266.

4. Gusyatiner O, Bady P, Pham MDT, Lei Y, Park J, Daniel RT*, et al.* BET inhibitors repress expression of interferon-stimulated genes and synergize with HDAC inhibitors in glioblastoma. *Neuro Oncol* 2021, **23**(10)**:** 1680-1692.

5. Milligan GW, Cooper MC. An examination of procedures for determining the number of clusters in a data set. *Psychometrika* 1985, **50**(2)**:** 159-179.

6. Lin X, Huang X, Uziel T, Hessler P, Albert DH, Roberts-Rapp LA*, et al.* HEXIM1 as a robust pharmacodynamic marker for monitoring target engagement of BET family bromodomain inhibitors in tumors and surrogate tissues. *Mol Cancer Ther* 2017, **16**(2)**:** 388-396.
