# Supplementary material for: BET protein inhibition sensitizes glioblastoma cells to temozolomide treatment by attenuating MGMT expression
Source: Cell Death Dis. 2022 Dec 13;13(12):1037. doi: 10.1038/s41419-022-05497-y (PMC9747918; doi:10.1038/s41419-022-05497-y)

Full WESTERN Blots – Tancredi et al

1. Biological replicate shown cropped in Figure 2B

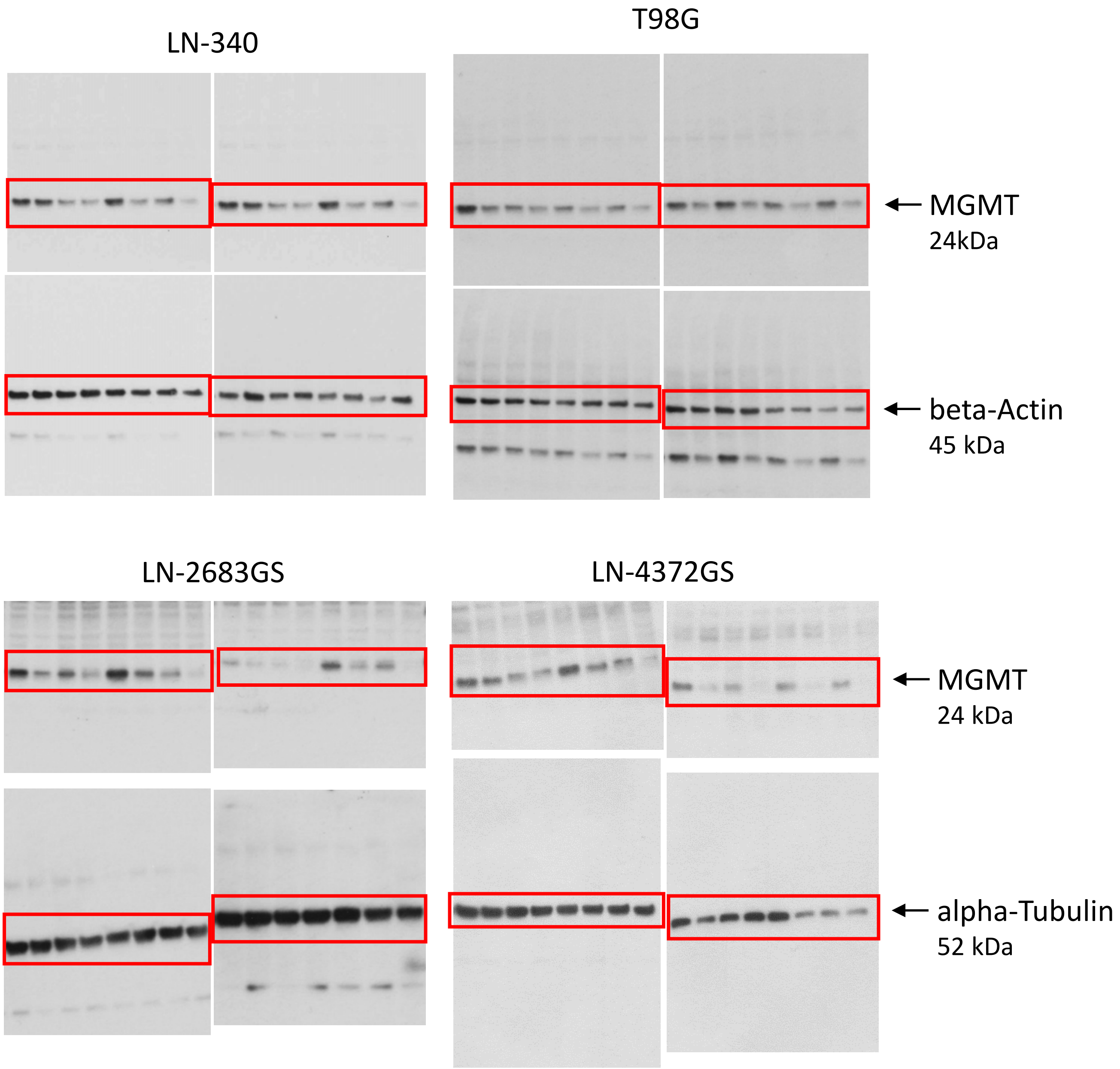

Description of samples and treatments

Time [hours]	24				48				72				120			
TMZ	-	-	+	+	-	-	+	+	-	-	+	+	-	-	+	+
JQ1	-	+	-	+	-	+	-	+	-	+	-	+	-	+	-	+

2. Second biological replicate for Figure 2B

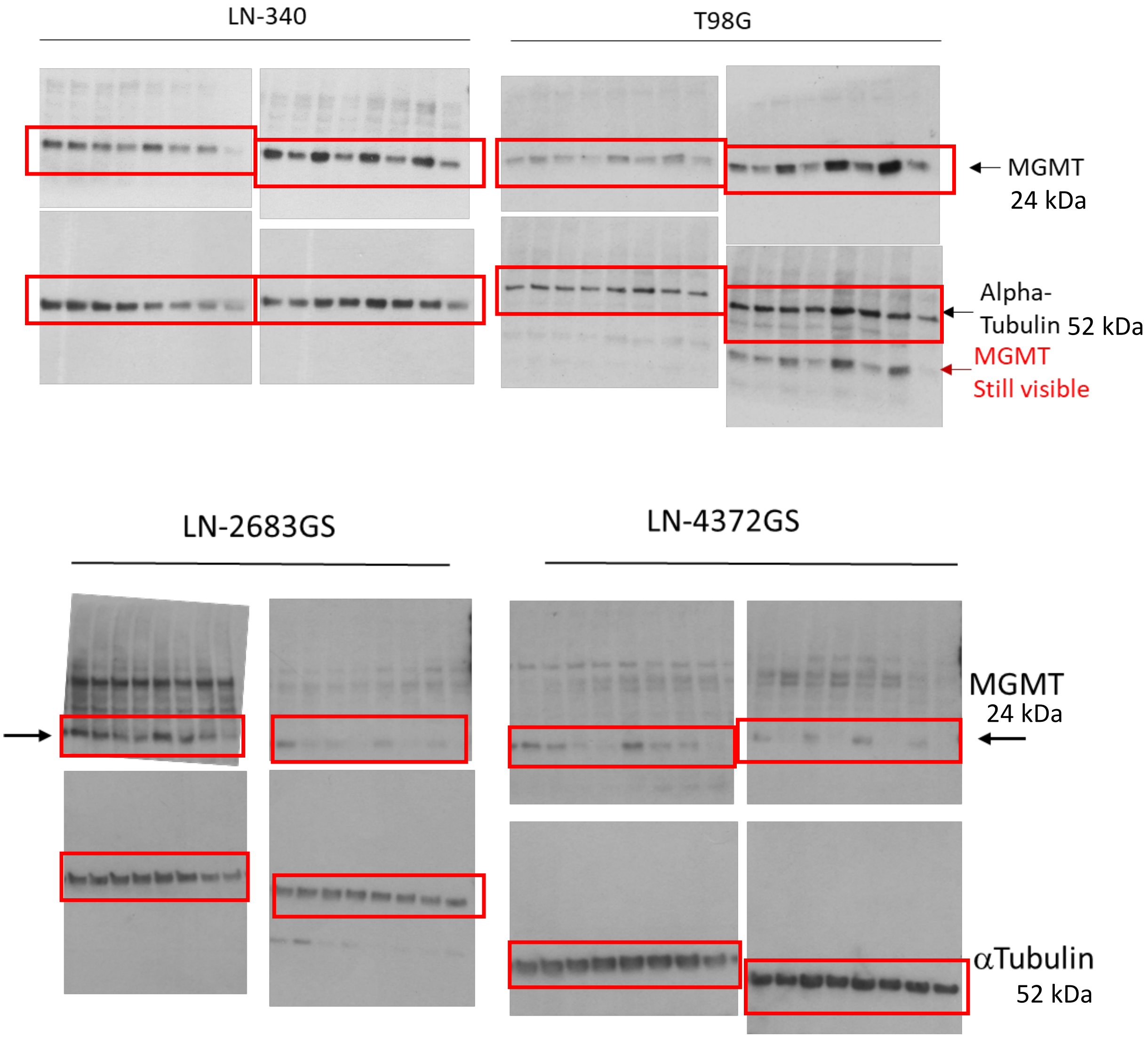

Description of samples and treatments

Time [hours]	24				48				72				120			
TMZ	-	-	+	+	-	-	+	+	-	-	+	+	-	-	+	+
JQ1	-	+	-	+	-	+	-	+	-	+	-	+	-	+	-	+

3. Biological replicate shown cropped in Figure 6A

LN-340

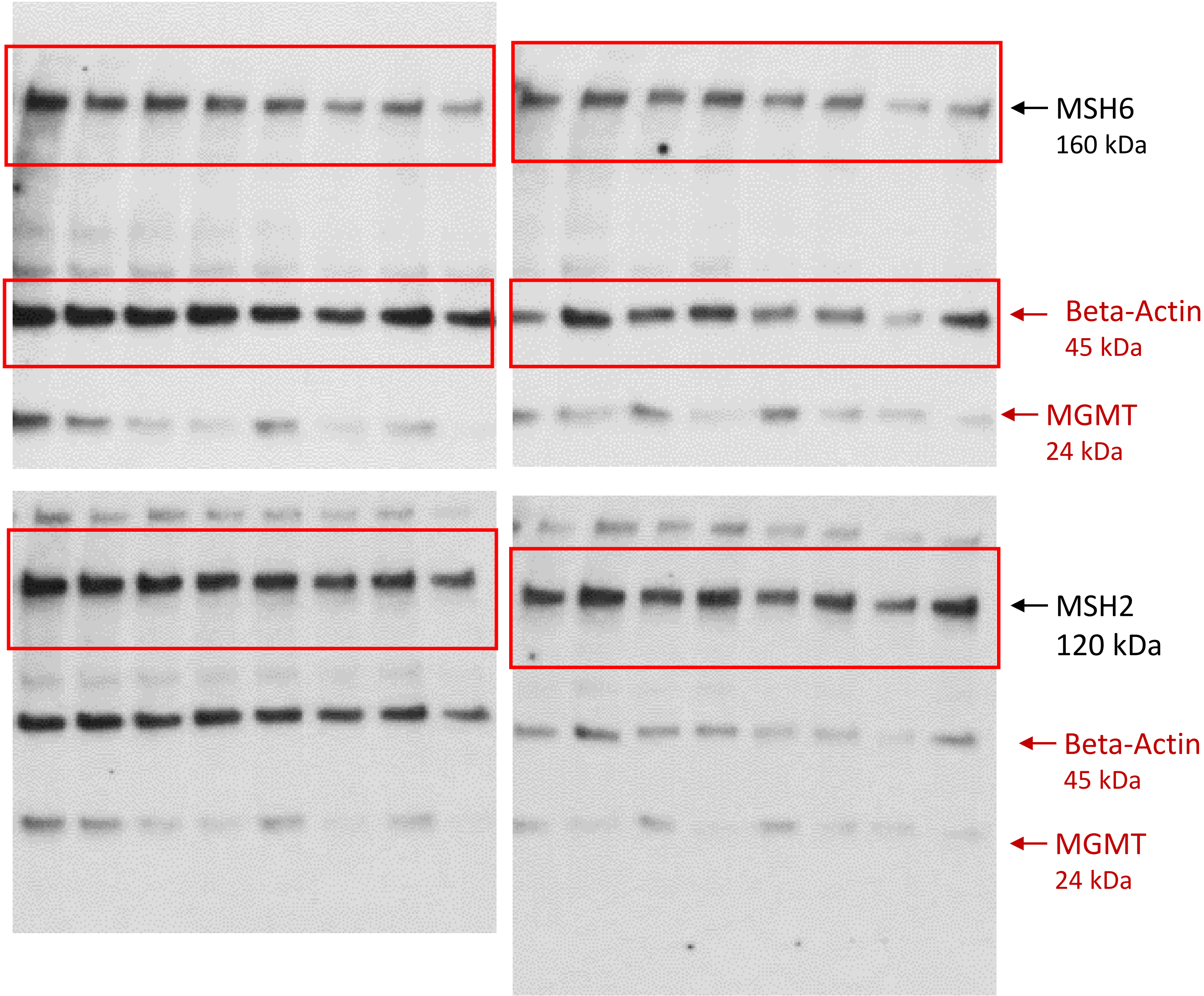

→ Same membrane from Figure 2B – MGMT and beta-Actin still visible

Description of samples and treatments

Time [hours]	24				48				72				120			
TMZ	-	-	+	+	-	-	+	+	-	-	+	+	-	-	+	+
JQ1	-	+	-	+	-	+	-	+	-	+	-	+	-	+	-	+

4. Biological replicate shown cropped in Figure 6D

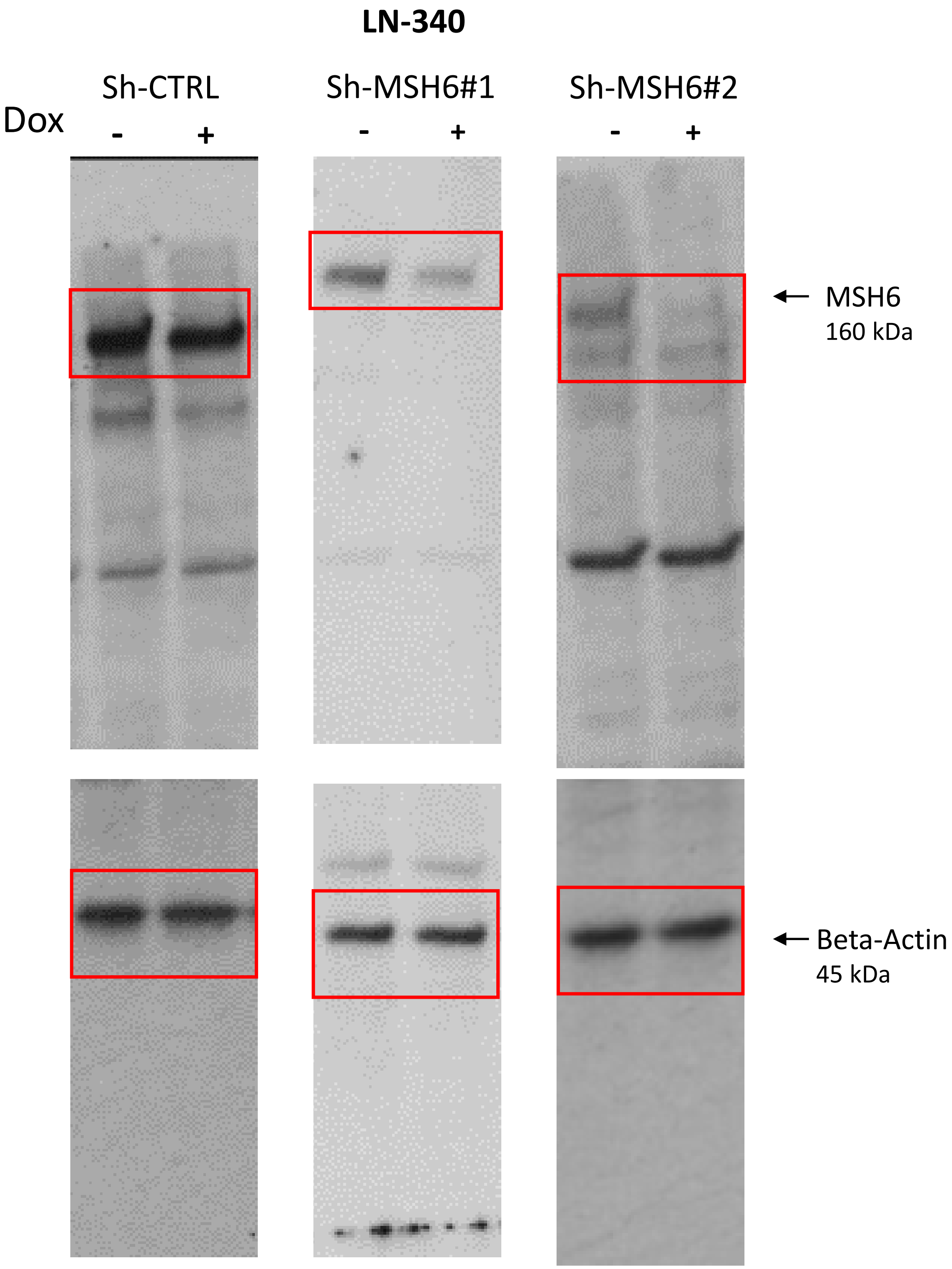

Supplement: Supplementary file 3 — Original Data File [file 41419_2022_5497_MOESM3_ESM.pdf]
